# Airway surface liquid acidification initiates host defense abnormalities in Cystic Fibrosis

**DOI:** 10.1038/s41598-019-42751-4

**Published:** 2019-04-24

**Authors:** Juliette Simonin, Emmanuelle Bille, Gilles Crambert, Sabrina Noel, Elise Dreano, Aurélie Edwards, Aurélie Hatton, Iwona Pranke, Bérengère Villeret, Charles-Henry Cottart, Jean-Patrick Vrel, Valérie Urbach, Nesrine Baatallah, Alexandre Hinzpeter, Anita Golec, Lhousseine Touqui, Xavier Nassif, Luis J. V Galietta, Gabrielle Planelles, Jean-Michel Sallenave, Aleksander Edelman, Isabelle Sermet-Gaudelus

**Affiliations:** 1 Inserm U1151 - CNRS UMR 8253 – Institut Necker Enfants Malades, Université Paris Sorbonne, Paris, France; 2 Centre de Recherche des Cordeliers, INSERM, Sorbonne Université, USPC, Université Paris Descartes, Université Paris Diderot, CNRS ERL 8228, Laboratoire de Physiologie Rénale et Tubulopathies, F-75006 Paris, France; 3 Inserm, UMR1152, PR CE Université Paris Diderot, Paris, France; 4 Department of Biomedical Engineering, Boston University, Boston, United States; 5 Upres EA2511, Université Paris Descartes, Institut Pasteur, Paris, France; 6 Telethon Institute of Genetics and Medicine, Pozzuoli, Italy

**Keywords:** Cell biology, Microbiology

## Abstract

Cystic fibrosis (CF) is caused by defective Cystic Fibrosis Transmembrane Conductance Regulator (CFTR) protein. Morbidity is mainly due to early airway infection. We hypothesized that *S. aureus* clearance during the first hours of infection was impaired in CF human Airway Surface Liquid (ASL) because of a lowered pH. The ASL pH of human bronchial epithelial cell lines and primary respiratory cells from healthy controls (WT) and patients with CF was measured with a pH microelectrode. The antimicrobial capacity of airway cells was studied after *S. aureus* apical infection by counting surviving bacteria. ASL was significantly more acidic in CF than in WT respiratory cells. This was consistent with a defect in bicarbonate secretion involving CFTR and SLC26A4 (pendrin) and a persistent proton secretion by ATP12A. ASL demonstrated a defect in *S. aureus* clearance which was improved by pH normalization. Pendrin inhibition in WT airways recapitulated the CF airway defect and increased *S. aureus* proliferation. ATP12A inhibition by ouabain decreased bacterial proliferation. Antimicrobial peptides LL-37 and hBD1 demonstrated a pH-dependent activity. Normalizing ASL pH might improve innate airway defense in newborns with CF during onset *of S. aureus* infection. Pendrin activation and ATP12A inhibition could represent novel therapeutic strategies to normalize pH in CF airways.

## Introduction

Cystic fibrosis (CF) is a lethal autosomal recessive disorder caused by mutations in the CF Transmembrane Conductance Regulator (*CFTR*) gene encoding for a cAMP-activated anionic channel, secreting mainly chloride (Cl^−^) and bicarbonate (HCO_3_^−^) at the apical surface of epithelia. Most patients are homozygous for p.Phe508del mutation (F508del). The main cause of morbidity and mortality is an obstructive lung disease^1,2^. This is characterized by exacerbated inflammation and bacterial infection of airway surface liquid (ASL), a thin layer coating the luminal face of the airway epithelium^3–6^.

ASL bacterial colonization begins within the first hours of life with evidence of *Staphylococcus aureus* (*S. aureus*) in airway secretions pointing to impaired local defense. However, the mechanism responsible for this defective bacterial clearance is not clearly understood. Proposed explanations relate to decreased mucociliary clearance^7^ and abnormal inflammatory responses^3^, but recent studies also suggested reduced antimicrobial capacity of CF ASL^1,8^. Studies in newborn pigs with CF highlighted an abnormally low ASL pH in association with defective short-term *S. aureus* antimicrobial activity^1^. Restoring normal pH improved the ability to eradicate the bacteria, suggesting that reduced ASL pH is central to disease pathogenesis^1^. An abnormally low ASL pH might indeed alter local antibacterial defense by impairing mucin hydration and solubilization, resulting in hyper-viscous mucus, which impedes mucociliary clearance^9–11^. It also reduces the activity of antimicrobial peptides by modulating their native charges^1,5,12–15^.

The value of ASL pH in patients with CF has been questioned in a recent study which showed similar ASL pH in young children with and without CF^16^. Most importantly, there is no clear understanding of the initial host response, when *S. aureus* bacteria infiltrate the pristine surface of the newborn airway, with prolonged time of contact and continuous reseeding from infected mucus plugs. It is therefore crucial to clarify the pathogenesis of these very early steps to counteract the pro-infectious vicious circle and to establish the optimal therapeutic strategy in newborns with CF.

We hypothesized that *S. aureus* clearance during the first hours of infection is impaired in human CF ASL because of lowered ASL pH. We designed bacterial infection experiments within human airway epithelia to mirror the onset of initial *S. aureus* infection. We then studied the relationship between local bacterial clearance and ASL pH in WT and F508del homozygous human bronchial epithelial cells, with a specific emphasis on physiologically relevant HCO_3_^−^ and protons (H^+^) transporters.

## Materials and Methods

### Human bronchial epithelial cell cultures

Immortalized CFBE41o^−^ bronchial epithelial cells, with expression of wild-type and F508del-CFTR were provided by Dr. Gruenert^17^. Human primary bronchial epithelial (HBE) cells were obtained from lobectomies of non-CF donors and lung explants of patients with CF after written informed consent from all the patients. Cells were differentiated and grown at air-liquid interface (ALI) for 3 to 4 weeks, as previously described^18^. All experiments were performed in accordance with the guidelines and regulations described by the Declaration of Helsinki and the Huriet-Serusclat and Jardet law on human research ethics. The study was approved by the Ile de France 2 Ethics Committee.

### Measurement of ASL pH with a microelectrode in a controlled atmosphere

To measure ASL pH reliably under physiological conditions, we designed a system with a controlled atmosphere enclosure allowing the regulation and monitoring of gas atmosphere, temperature and hygrometry to maintain physiological ASL conditions (Tech Systèmes, Ris-Orangis, Ile-de-France, France). This enclosure enabled to maintain pCO_2_ at 5% and temperature at 37 °C.

pH was measured in the controlled enclosure with a micro-combination pH electrode (Thermo Scientific Orion 9810BN, Illkirch, Grand Est, France). The pH microelectrode was calibrated before each experiment with buffer at pH 4 and 7. In a preliminary validation study, we measured the pH values of Ringer’s solutions containing 10 or 25 mM HCO_3_^−^ in the controlled enclosure at 5% pCO_2_ and 37 °C after 2 hours incubation. We checked that in this set-up, the measured pH value did not differ by more than 0.03 pH unit from the theoretical one, calculated according to the Henderson Hasselbalch equation, i.e pH = 7.4 for 25 mM HCO_3_ and pH = 7.1 for 10 mM HCO_3_.

As the measurements obtained by positioning the pH microelectrode directly in contact to the epithelium were not reproducible, possibly because of epithelium disruption, measurements required the addition at the apical side of the cell culture of 50 µl Ringer’s solution, this volume being the minimal volume to homogeneously cover the filter, and allow reliable measurements.

Respiratory cell cultures were bathed at the basal face with culture medium displaying a 25 mM HCO_3_^−^ concentration. The solution added at the apical face was a Ringer’s solution (115 mM NaCl, 25 mM NaHCO_3_, 2.4 mM K_2_HPO_4_, 0.4 mM KH_2_PO_4_, 1.2 mM CaCl_2_, 1.2 mM MgCl_2_ and 10 mM Glucose), whose pH was equilibrated beforehand with 25 mM HCO_3_^−^ in 5% CO_2_. This 50 µL solution, representing diluted ASL, was recovered after 15 minutes to 6 hours incubation. pH was measured immediately, directly in the enclosure with a micro-combination pH-electrode. To investigate pH regulation, cell cultures were incubated with a 10 mM HCO_3_^−^ solution in 5% CO_2_ (see supplemental material for composition), to mimic a moderate extracellular normocapnic acidosis or with agonists or inhibitors of targeted transporters.

### Short-circuit current measurement

Short-circuit current (SCC) was measured under a voltage clamp with an EVC4000 Precision V/I Clamp (World Precision Instruments, Sarasota, Florida, United States). Culture inserts with differentiated bronchial epithelial cells were mounted in Ussing chambers (Physiologic Instruments, San Diego, California, United States). Experiments were performed at 37 °C, by bubbling epithelia with a pCO_2_ = 5% and a pO_2_ = 95%.

For measurements of transepithelial HCO_3_^−^ secretion, SCC experiments were conducted in an apical and basolateral solution without Cl^−^ and with 25 mM HCO_3_^−^ (125 mM Na isethionate, 25 mM NaHCO_3_, 2.4 mM K_2_HPO_4_, 0.6 mM KH_2_PO_4_, 3.0 mM Ca-gluconate, 2.4 mM Mg-gluconate and 10 mM Dextrose, pH 7.4). During continuous recording of SCC, the following inhibitors and activators were added: the ENaC-channel blocker amiloride (100 µM), cAMP agonists forskolin (10 µM) and IBMX (100 µM) to activate the transepithelial cAMP-dependent current, VX-770 (10 µM) to potentiate CFTR activity, and the specific CFTR inhibitor Inh-172 (5 µM).

### Quantitative reverse transcription polymerase chain reaction (qRT-PCR) analysis

qRT-PCR were performed following standard protocols (See Supplemental Material and Supplemental Tables 1 and 2).

### ASL antibacterial growth capacity study

*S. aureus* CIP 76.25 (Collection of lnstitut Pasteur) was cultured in antibiotic-free cell culture medium. Epithelia were apically infected with 50 µL of different inocula. ASL was collected at 2 or 6 hours after incubation at 5% CO_2_, 37 °C, and plated on Petri dishes. Surviving bacteria were evaluated by counting colony-forming units (CFUs) and expressed as % from inoculum or control condition. To determine effect of ASL pH on bacterial growth, bacterial culture medium was set at various pH by modifying HCO_3_^−^ concentrations or incubated for 6 hours with 25 µM specific SLC26A4 (pendrin) inhibitor A01^19^ or 1 mM ATP12A (H^+^/K^+^ ATPase) inhibitor ouabain and infected for 2 hours with *S. aureus* CIP 76.25. *S. aureus* CIP 76.25 adhesion and internalization capacities on airway epithelium were evaluated by CFU counting and immunostaining^20^ (See Supplemental Material).

### LL-37 and hBD1 antimicrobial peptides antimicrobial capacity study at different pH

*S. aureus* CIP 76.25 was cultured in antibiotic-free cell culture medium. Bacterial cultures were initiated with OD_600 nm_ = 0.05 and left under agitation at 37 °C, 5% CO_2_.

To determine the impact of pH change on LL-37 (Invivogen, San Diego, California, United States) or on hBD1 (Eurogentec, Liège, Bruxelles) antimicrobial capacity, bacterial cultures were diluted 2-fold (final inoculum 100,000 CFU/mL) with Ringer’s solutions containing the required HCO_3_^−^ concentrations to get at pCO_2_ 5% and at 37 °C the following pH values: 7.00, 7.40 and 7.70.

A stock solution of 1 mg/mL LL-37 or hBD1 was prepared in sterile water with 0.02% acetic acid and 0.4% BSA (bovine serum albumin); 5 µL of the LL-37 stock was added to 95 µL of inoculum to obtain a final LL-37 or hBD1 concentration of 50 µg/mL for 2 hours incubation at 5% CO_2_, 37 °C. Solutions were collected after the 2 hours incubation and plated on Petri dishes. Surviving bacteria were evaluated by CFU counting.

### Statistical analysis

Data are presented as mean ± standard error of mean (SEM) of each experiment. Statistical significance was assessed by non-parametric Wilcoxon test, Kruskal Wallis test or t-test when appropriate; *p* ≤ 0.05 was considered statistically significant. Data were analyzed with StatView software.

All data generated or analysed during this study are included in this published article (and its Supplementary Information files).

## Results

### pH is abnormally low in the ASL of CF bronchial epithelial cells

**Figure 1:**
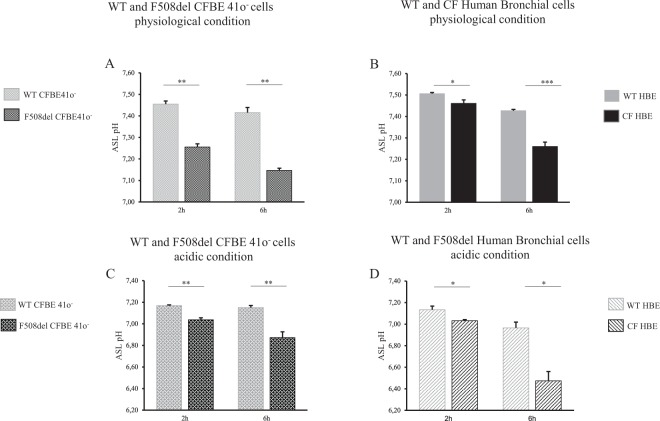
Airway surface liquid pH measurements under physiological and normocapnic acidosis conditions in WT and F508del bronchial epithelia at 2 and 6 hours. Apical fluid pH was measured after apical addition for 2 hours or 6 hours of 50 µL of physiologic Ringer’s solution (25 mM HCO_3_^−^, 5% CO_2_) (upper panel) or acidic Ringer’s (10 mM HCO_3_^−^, 5% CO_2_) (lower panel). (**A**) WT *vs*. F508del CFBE41o^−^ cells. 2 h: pH = 7.45 ± 0.01 *vs*. pH = 7.26 ± 0.01, *p* = 0.008; 6 h: pH = 7.42 ± 0.02 *vs*. pH = 7.15 ± 0.01, *p* = 0.008. (**B**) WT *vs*. CF Human Bronchial Epithelial (HBE) primary cells. 2 h: pH = 7.51 ± 0.004 *vs*. pH = 7.46 ± 0.02, *p* = 0.036; 6 h: pH = 7.43 ± 0.006 *vs*. pH = 7.26 ± 0.02, *p* < 0.001. (**C**) WT *vs*. F508del CFBE41o^−^ cells. 2 h: pH = 7.17 ± 0.01 *vs*. pH = 7.04 ± 0.02, *p* = 0.008; 6 h: pH = 7.15 ± 0.02 *vs*. pH = 6.87 ± 0.05, *p* = 0.008. (**D**) WT *vs*. F508del HBE primary cells. 2 h: pH = 7.13 ± 0.03 *vs*. pH = 7.03 ± 0.01, *p* = 0.028; 6 h: pH = 6.97 ± 0.05 *vs*. pH = 6.47 ± 0.09, *p* = 0.028. For all conditions, n = 3 in triplicate except for figure B where CF bronchial primary cells were derived from 3 F508del homozygote donors (triplicate for each patient) and 1 N1303K/4005 + 1G > A patient (12 filters). Data are presented as mean ± SEM. Statistical significance from unpaired nonparametric Wilcoxon test. *p < 0.05; **p < 0.01; ***p < 0.001; NS: non significant.

In this experimental series, ASL pH was measured at various time intervals after the addition of 50 µL of physiological Ringer’s solution (25 mM HCO_3_^−^, 5% CO_2_) at the apical face of CFBE41o^−^ cell lines and primary cells from 3 F508del homozygote donors and 1 N1303K/4005 + 1G > A patient (Fig. 1A,B). The pH was significantly reduced in CF cells compared to WT cells. This difference was apparent from 15 minutes after addition of Ringer’s solution (Supplemental Fig. 1A) and was significant at 2 hours and 6 hours of incubation in cell lines and primary cells.

Cells were then subjected to extracellular normocapnic acidosis (10 mM HCO_3_^−^, 5% CO_2_). As expected, ASL pH decreased in both WT and F508del CFBE41o^−^ cell lines, and the ASL pH of CF cultures remained significantly lower than the ASL pH of WT cells (Fig. 1C,D; Supplemental Fig. 1B). These results allowed us to determine that, in physiological conditions, there was a net proton secretion flux in the F508del cells averaging 1.8 µeq/hr/cm^2^ at 15 minutes in the F508del CFBE41o^−^ cell lines and 4 µeq/hr/cm^2^ in the F508del HBE cells. Both values are significantly higher than corresponding fluxes in WT cells (See Supplemental results, Supplemental Table 3).

### ATP12A-mediated H+ secretion is present in CF and non-CF bronchial epithelial cells

Transcripts of the non-gastric H^+^/K^+^ adenosine triphosphatase (ATPase) ATP12A, were detected in CFBE41o^−^ and bronchial primary cells of both WT and F508del genotypes (Supplemental Fig. 2).

**Figure 2:**
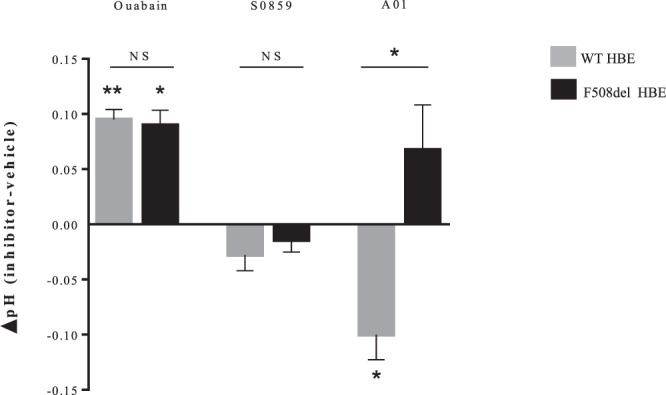
Airway surface liquid pH measurements upon ATP12A, NBC1 and SLC26A4 activity inhibition in WT and F508del Human Bronchial Epithelial primary cells. pH changes (ΔpH) in Human Bronchial Epithelial (HBE) primary cells after incubation with vehicle or specific inhibitor are shown in WT HBE cells (grey bars) or F508del HBE (black bars). Apical fluid pH was measured after apical addition of 50 µL of physiologic Ringer’s solution (25 mM HCO_3_^−^, 5% CO_2_) for 2 hours. Ouabain 1 mM (or Ringer as vehicle) was added at the apical side for 2 hours, as ATP12A inhibitor; S0859 100 µM (or DMSO as vehicle) was added at the basal side for 2 hours, as NBC1 inhibitor; A01, 25 µM (or DMSO as vehicle) was added at the basal side for 6 hours, as SLC26A4 inhibitor. Ouabain. WT HBE: ΔpH (Ouabain-Ringer) = 0.095 ± 0.009 (*p* = 0.002); F508del HBE: ΔpH = 0.09 ± 0.013 (*p* = 0.028); WT *vs*. F508del HBE ΔpH: NS. For all conditions, n = 4 in triplicate. S0859. WT HBE: ΔpH (S0859-DMSO) = −0.02 ± 0.014 (NS); F508del HBE: ΔpH = −0.02 ± 0.011 (NS); WT *vs*. F508del HBE ΔpH: NS. For all conditions, n = 3 in triplicate. A01. WT HBE ΔpH (A01-DMSO) = −0.10 ± 0.022 (*p* = 0.043); F508del HBE ΔpH = 0.068 ± 0.041 (NS); WT *vs*. F508del HBE ΔpH: *p* = 0.04. For all conditions, n = 3 in triplicate. Data are presented as mean ± SEM. Statistical significance from unpaired nonparametric Wilcoxon test. Level of significance is shown for ΔpH in each genotype as well as the comparison between genotypes. *p < 0.05; **p < 0.01; NS: non significant.

To evaluate the contribution of ATP12A to ASL pH, 1 mM ouabain was applied on the apical side of the epithelium to selectively inhibit apical ATP12A^8^. In physiological conditions, ouabain induced a significant alkalinization of the apical fluid both in WT and in F508del HBE (Fig. 2). This increase (~0.1 pH unit) was similar in both genotypes. WT and F508del HBE were then incubated at the basal side in a nominally CO_2_/HCO_3_^−^ free solution which was buffered at pH 7.4 with Hepes/NaOH and at the apical side in a nominally CO_2_/HCO_3_^−^ free, Hepes free-solution whose pH equilibrated at 7.25. In these conditions, ouabain induced a significant increase in ASL pH in both WT (ΔpH = 0.15; p = 0.01) and F508del CFBE41o^−^ (ΔpH = 0.12; p = 0.01) (Supplemental Fig. 3**)**. This increase in both genotypes was similar to that obtained in physiological conditions.

### HCO_3_^−^ secretion is defective in CF epithelial cells and involves CFTR and SLC26A4

Since inhibition of ATP12A-dependent H^+^ secretion did not fully restore CF ASL pH in CF airway epithelium, we evaluated the contribution of HCO_3_^−^ transport impairment to explain abnormal ASL pH.

Transcripts of the basolateral Cl^−^/HCO_3_^−^ exchanger AE2, the Na^+^/HCO_3_^−^ co-transporter NBC1, and the apical Cl^−^/HCO_3_^−^ exchanger pendrin were detected by qRT-PCR. AE2 transcript level was not significantly different between WT and F508del CFBE41o^−^. Pendrin and NBC1 transcripts were significantly more elevated in F508del CFBE41o^−^ or HBE *vs*. WT cells (Supplemental Fig. 2).

To evaluate the contribution of HCO_3_^−^ transepithelial secretion to apical pH, we first investigated the basolateral HCO_3_^−^ uptake. Selective inhibition of NBC1 did not significantly modify ASL pH neither in WT nor in F508del HBE (Fig. 2). NBC1 inhibition slightly decreased ASL pH in WT CFBE41o^−^ cell lines, but there was no change in F508del CFBE41o^−^ (Supplemental Fig. 4).

**Figure 3:**
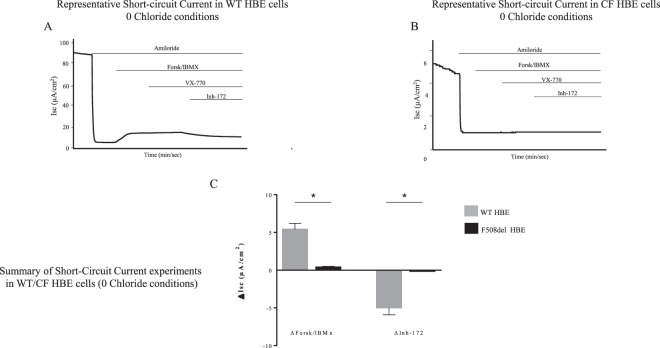
Short-circuit current measurements under 0 Chloride conditions in WT and CF Human Bronchial Epithelial primary cells. Experiments were done in a solution with 0 Cl^−^ and 25 mM HCO_3_^−^, bubbled with 95% O_2_ and 5% CO_2_ at the apical and basal faces. Short-circuit current variation were induced by successive addition of amiloride (100 µM), Forskolin/IBMX (10 µM/100 µM), VX770 (10 µM), Inh-172 (5 µM). (**A**) Representative experiment from WT Human Bronchial Epithelial (HBE) primary cells. (**B**) Representative experiment from CF HBE primary cells (N1303K/4005 + 1G > A). (**C**) Summary of results obtained from 3 WT subjects (n = 3; 14 filters) (grey bars) and 1 CF patient (N1303K/4005 + 1G > A; 2 independent experiments; 7 filters) (black bars). ΔForsk/IBMX: 5.4 ± 0.8 µA/cm^2^ in WT HBE *vs*. 0.4 ± 0.1 /cm^2^ in CF HBE; *p* = 0.012. ΔInh-172: −4.9 ± 0.97 µA/cm^2^ in WT HBE *vs*. −0.1 ± 0.05 µA/cm^2^ in F508del HBE; *p* = 0.02. Data are presented as mean ± SEM. Statistical significance from unpaired nonparametric Wilcoxon test. *p < 0.05.

We then investigated the apical transporters involved in HCO_3_^−^ secretion. The CFTR, cAMP-dependent, HCO_3_^−^ transport was evaluated by short-circuit current (SCC) in a solution depleted of Cl^−^. In these conditions, the CFTR-dependent SCC signal is due to the active transport of anions other than Cl^−^, mainly HCO_3_^−^. There was a significant response to forskolin/IBMX in WT primary cells, with a further inhibition by the CFTR inhibitor Inh-172. By contrast, in F508del homozygous primary cells, cAMP stimulation did not induce any HCO_3_^−^ transport (Fig. 3).

Neither CFTR activation by the forskolin/IBMX cocktail, even under extracellular normocapnic acidosis conditions (Supplemental Fig. 5), nor addition of the specific CFTR inhibitor PPQ-102 (2 µM) (Supplemental Fig. 6), significantly modified ASL pH in the WT CFBE41o^−^ cells. CFTR inhibition by Inh-172, after its preactivation by forskolin and IBMX, induced a significant pH decrease in normocapnic acidosis conditions, showing a significant involvement of CFTR in pH regulation (Supplemental Fig. 7). However, the fact that this pH modulation is observed only in non-physiological acidic conditions, and most importantly, that CFTR activation is not sufficient to restore pH upon mild acidosis (Supplemental Fig. 5) points to the role of other transporters in ASL pH regulation.

**Figure 4:**
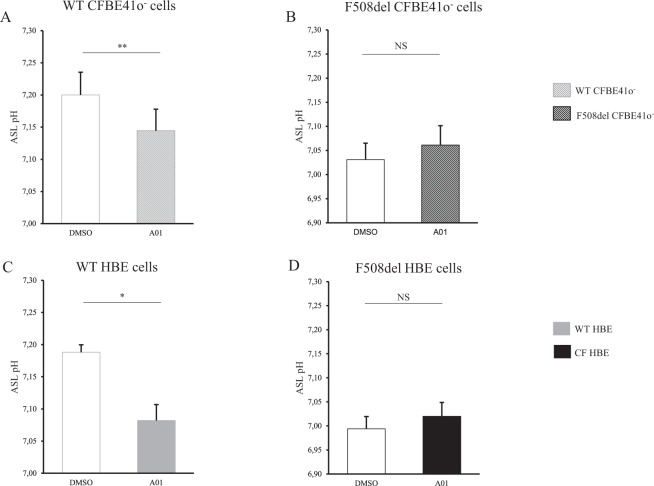
Airway surface liquid pH measurements upon SLC26A4 inhibition in WT and F508del bronchial epithelia. CFBE41o^−^ cells and Human Bronchial Epithelial (HBE) primary cells were incubated with DMSO vehicle (empty bars) or specific pendrin inhibitor A01 (filled bars) for 6 h. ASL pH was measured after apical addition of 50 µL Ringer’s solution (25 mM HCO_3_^−^, 5% CO_2_). DMSO induces a slight ASL acidification in both WT and F508del cells reaching a decrease of 0.2 pH units after 6 h incubation. (**A**) WT CFBE41o^−^ cells. pH = 7.20 ± 0.04 (DMSO) *vs*. pH = 7.14 ± 0.03 (A01), *p* = 0.008. (**B**) F508del CFBE41o^−^ cells. pH = 7.03 ± 0.03 (DMSO) *vs*. pH = 7.06 ± 0.04 (A01), NS. (**C**) WT HBE primary cells. pH = 7.19 ± 0.01 (DMSO) *vs*. pH = 7.08 ± 0.02 (A01), *p* = 0.04. (**D**) F508del HBE primary cells. pH = 6.99 ± 0.03 (DMSO) *vs*. pH = 7.02 ± 0.03 (A01), NS. For all conditions, n = 3 in triplicate. Data are presented as mean ± SEM. Statistical significance from unpaired nonparametric Wilcoxon test. *p < 0.05; **p < 0.01; NS: non significant.

Inhibition of pendrin activity with its specific inhibitor A01 induced a significant ASL acidification in both WT CFBE41o^−^ cell lines and primary cells but not in F508del cells (Figs 2 and 4).

Pendrin inhibition by A01 was associated with a significant decrease in the SCC variation in WT HBE cells in response to forskolin/IBMX and Inh-172 (Supplemental Fig. 8). These effects were not detected in F508del cell lines and primary cells (Figs 2 and 4).

### *S. aureus* clearance is defective in ASL of CF respiratory epithelium and is pH-dependent

**Figure 5:**
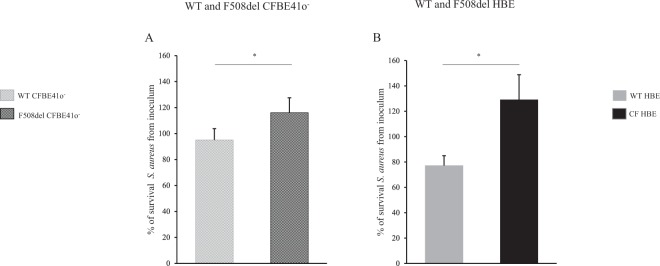
Clearance of *S. aureus* CIP 76.25 in airway surface liquid of WT and F508del bronchial epithelia. Epithelia were apically infected with *S. aureus* CIP 76.25 (3,000 CFU/mL). ASL was collected after 2 hours and plated on Petri dishes to count the surviving bacteria expressed as % from the inoculum. (**A**) CFBE41o^−^ cells. % of surviving bacteria at 2 h: 95 ± 9% in WT *vs*. 116 ± 11% in F508del, *p* = 0.04. For all conditions, n = 5 in triplicate. (**B**) Human Bronchial Epithelial (HBE) primary cells. % of surviving bacteria at 2 h: 77 ± 8% in WT (n = 9 in triplicate) *vs*. 129 ± 20% in F508del (n = 3 in triplicate), *p* = 0.04. Data are presented as mean ± SEM. Statistical significance from unpaired nonparametric Wilcoxon test. *p < 0.05.

We first determined the optimal conditions for infection by finding a middle ground between intense bacterial proliferation overwhelming the cells and too low or too short infection period that would not allow us to detect any difference (Supplemental Fig. 9). Those experimental conditions were meant to reflect the bacterial exposition of human CF airways to the environment. As a whole, primary cells displayed a more potent antimicrobial capacity against *S. aureus* than did CFBE41o^−^. This is illustrated by the significant decrease in the number of surviving bacteria after 2 h infection with 3,000 CFU/mL *S. aureus* in WT primary cells, whereas it remained almost stable in WT CFBE41o^−^ (Fig. 5).

Bacterial clearance by ASL was significantly decreased in CF cells *vs*. WT bronchial epithelial cells. This was observed at 2 hours post-infection in the CFBE41o^−^ cell line and in the primary cells: the percentage of surviving bacteria in CF *vs*. WT ASL increased 1.5-fold in CFBE41o^−^ cells (Fig. 5A) and 1.7-fold in primary cells (Fig. 5B) after infection with 3.000 CFU/ml. Supplemental Fig. 10 provides bacterial clearance results in conditions maximized to evidence the difference between the WT and the F508del cultures, *e.g*; 300 CFU/ml 2 h for CFBE41o^−^ and 3,000 CFU/ml 6 hours for primary cells. At 6 hours post-infection with 3,000 CFU/ml, the difference in the primary cell model increased 20-fold (~1.5-fold log increase). We verified that the WT *vs*. CF difference was not related to a differential *S. aureus* adhesion or internalization capacity between the WT and CF airway epithelia (Supplemental Fig. 10).

**Figure 6:**
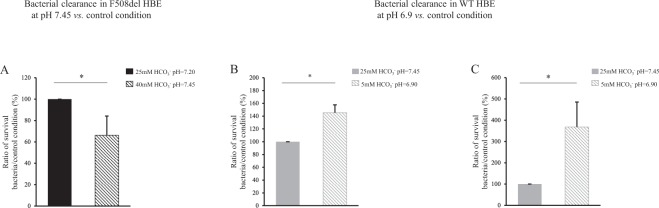
Effect of airway surface liquid pH on clearance of *S. aureus* CIP 76.25 in WT and F508del Human Bronchial Epithelial primary cells. Human Bronchial Epithelial (HBE) primary cells were apically infected with *S. aureus* CIP 76.25 (3,000 CFU/ml). The apical fluid was collected at the end of the experiments and plated on Petri dishes to count surviving bacteria. To investigate the effect of pH on bacterial clearance, pH of inoculum medium was changed by different HCO_3_^−^ concentrations. In WT HBE cells, HCO_3_^−^ concentrations at 5 mM and 25 mM corresponded respectively to a pH of apical supernatant at 6.90 and 7.45. In F508del HBE cells, HCO_3_^−^ concentrations at 25 mM and 40 mM corresponded respectively to a pH at 7.20 and 7.45. Surviving bacteria detected at the end of experiment in apical fluid under test conditions are expressed as ratio (%) to the control condition of 25 mM HCO_3_^−^ inoculum solution. (**A**) F508del HBE cells. % of surviving bacteria after 2 hours in 40 mM HCO_3_^−^ condition *vs*. 25 mM HCO_3_^−^ control condition: 66 ± 18%, *p* = 0.02. For all conditions, n = 3 in triplicate. (**B**) WT HBE cells. % of surviving bacteria after 2 hours in 5 mM HCO_3_^−^ condition *vs*. 25 mM HCO_3_^−^ control condition: 145 ± 12%, *p* = 0.01. For all conditions, n = 3, 4 filters per experiment. (**C**) WT HBE cells. % of surviving bacteria after 6 hours in 5 mM HCO_3_^−^ condition *vs*. 25 mM HCO_3_^−^ control condition: 368 ± 117%, *p* = 0.01. For all conditions, n = 3 in triplicate. Data are presented as mean ± SEM. Statistical significance from unpaired nonparametric Wilcoxon test. *p < 0.05.

**Figure 7:**
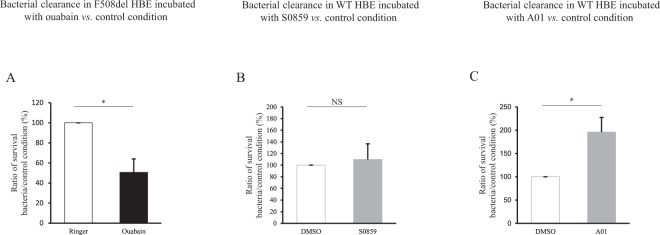
Effect of pH pharmacological modulation on clearance of *S. aureus* CIP 76.25 in airway surface liquid of WT and F508del Human Bronchial Epithelial primary cells. Human Bronchial Epithelial (HBE) primary cells were apically infected with *S. aureus* CIP 76.25 (3,000 CFU/ml) for 2 hours previously incubated with specific transporter inhibitors or vehicle. The apical fluid was collected at the end of the experiments and plated on Petri dishes to count surviving bacteria. Bacteria detected under test conditions were expressed as ratio to the control condition. (**A**) F508del HBE cells incubated with ouabain 1 mM, as ATP12A inhibitor *vs*. Ringer for 2 hours. % of surviving bacteria from inoculum: 50.6 ± 13%, *p* = 0.046. For all conditions, n = 2 in triplicate. (**B**) WT HBE cells incubated with S0859 100 µM, as NBC inhibitor, *vs*. DMSO for 2 hours. % of surviving bacteria from inoculum: 109.7 ± 27%, NS. For all conditions, n = 2 in triplicate. (**C**) WT HBE cells incubated with A01 25 µM, as SLC26A4 inhibitor, *vs*. DMSO for 6 hours. % of surviving bacteria from inoculum: 196 ± 32%, *p* = 0.028. For all conditions, n = 2 in triplicate. Data are presented as mean ± SEM. Statistical significance from unpaired nonparametric Wilcoxon test. *p < 0.05; NS: non significant.

We next investigated whether pH variations could modulate bacterial clearance capacity by varying HCO_3_^−^ concentrations at constant pCO_2_, 5%. In CF epithelia, increasing inoculum medium HCO_3_^−^ concentration to 40 mM normalized infected apical fluid pH from 7.20 to 7.45. This was associated with an improved antimicrobial capacity as evinced by a significant decrease in bacterial survival in primary cells (Fig. 6A). Pharmacological modulation of ASL pH by ouabain in F508del primary cells, which tends to normalize pH, was associated with a significant 2-fold decrease in bacterial proliferation (Fig. 7A).

In WT cells, decreasing the pH of the apical supernatant to 6.9 (5 mM HCO_3_^−^) significantly increased the number of surviving bacteria at 2 hours (Fig. 6B) and 6 hours (Fig. 6C). Inhibition of NBC1 activity did not induce any change in ASL antimicrobial capacity (Fig. 7B). In contrast, application of pendrin inhibitor A01 was associated with a significant overgrowth of *S. aureus* in primary cells (Fig. 7C) as well as in WT CFBE41o^−^ (Supplemental Fig. 12). Control experiments checked that neither pH change, nor ouabain, nor A01 affected bacterial growth (data not shown).

### pH-dependent LL-37 and hBD1 antimicrobial capacities against *S. aureus*

To investigate whether enhancement of bacterial proliferation in CF airways was related to a defect in the antimicrobial peptides hBD1, hBD2 and LL-37, we first measured their expression (mRNA) and showed no difference in either CFBE41o^−^ cells or primary cells independently of the genotype (WT or F508del) (Supplemental Fig. 13). Transcript levels did not change with pH when pH variations were similar to those observed between CF and WT epithelia (Supplemental Fig. 14).

**Figure 8:**
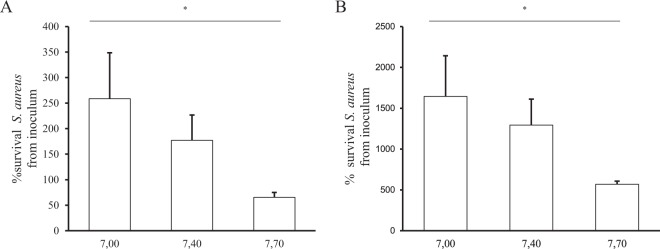
LL-37 and hBD1 antimicrobial capacities against *S. aureus* CIP 76.25 at different pH. LL-37 and hBD1 antimicrobial peptides were incubated at 50 µg/mL for 2 h with 100,000 CFU/mL of *S. aureus* CIP 76.25 in culture medium at pH = 7,00; 7.40 and 7.70. Solutions were plated at the end of the experiments on Petri dishes to count the surviving bacteria. Surviving bacteria are expressed as % from the inoculum under different conditions. (**A**) LL-37 activity evaluated at: pH = 7.00 (3 different cultures in duplicate), 7.40 (2 different cultures in duplicate) and 7.70 (n = 3 in duplicate), *p* = 0.01. (**B**) hBD1 activity evaluated at: pH = 7.00, 7.40 and 7.70, *p* = 0.03. For all conditions, n = 2 in duplicate. Data are presented as mean ± SEM. Statistical significance from unpaired nonparametric Kruskal-Wallis test. *p < 0.05.

We then tested how modification of pH may affect the activity of these antimicrobial peptides. As shown in Fig. 8A,B, LL-37 and hBD1 displayed significant pH-dependent antimicrobial activity at small pH variations, comparable to ASL pH differences observed between WT and CF epithelia.

## Discussion

Our findings demonstrate that ASL pH is significantly lower in primary cultures of airway cells obtained from patients with CF than those from healthy controls. This abnormal acidification is related to a defect in cAMP-dependent HCO_3_^−^ secretion involving both CFTR and pendrin, concomitant with persistent H^+^ secretion by ATP12A. Our experiments confirm that ASL pH is a key factor for *S. aureus* clearance from airways and that small pH variations, similar to those observed between CF and WT, modulate LL-37 and hBD1 antimicrobial peptide activity. Very importantly, our data show that inhibition of pendrin activity in WT airways recapitulates both the CF acidification and antimicrobial defect and that inhibition of ATP12A activity in CF epithelia improves pH regulation and significantly improves bacterial clearance.

### Technical challenges

To face the technical challenge of measuring pH in epithelial cell culture models, measurements were made under steady-state conditions to mirror the *in vivo* airway microenvironment. This was achieved by using a humidified enclosure to avoid evaporation of the small ASL volume and allow control of CO_2_ content and temperature.

pH measurements were performed after at least 2 hours of incubation, as a compromise between the conditions needed for bacterial measurements (risk of bacterial overgrow after 2 hours) and the delay necessary for pH equilibration.

Because of the above limitations, instead of considering absolute values, we assessed the changes in the pH of this apical fluid and assumed that the latter reflected the changes in ASL pH. Therefore, our experimental strategy focused on the comparison between the 2 genotypes. Finally, because pH variations can not discriminate between H^+^ and HCO_3_^−^ transport we used pharmacological modulation of targeted transport to investigate the pathways of pH regulation in ASL.

### ASL pH regulation in CF airways and considerations on the specific role of pendrin

In line with previous studies^21,22^, we found that the ASL pH of CF epithelium was lower than that of WT epithelium, both in the F508del CFBE41o^−^ cell line and in HBE primary cells from patients. Importantly, CF cells could not compensate for acidosis, in contrast to WT cells, suggesting a defect in pH homeostasis in CF.

Other studies, however, reported that airway surface is not more acidic in patients with CF^23,24^. For example, a recent study based on the use of a pH-sensitive luminescent dye-based fiber-optic probe *in vivo* did not show any pH difference between CF and non-CF infants^16^. Those differences might be related to the methodology (fiber optic probe *vs*. microelectrode), the model used (*in vivo*, during breathing *vs*. primary culture devoid of any shear stress) but also the time of measurements, as they were performed at steady state in our study *vs*. immediately after positioning of the probe in the above study.

Importantly in the other study^16^, the mean age of the children was 50 (12–78) months, an age where the disease has probably already evolved, including abnormal mucus production and initiation of pro-inflammatory conditions, which might modify HCO_3_^−^ and H^+^ transporter expression levels or activity. In contrast, our model evaluates ASL virtually free of any inflammation and mucus, which very probably sets a different physio-pathological context and possibly alters transporter function. Although it can be argued that our model is artificial, we point out that it reflects the condition of the pristine neonate airways, devoid of any infection and probably inflammation-free, to best unravel the defects favoring the initial *S. aureus* colonization, according to our study’s hypothesis. This might therefore explain the resultant difference and accords with recent findings in the pig model where pH is lower in CF piglets at birth but tends to normalize after 3 weeks of life^25^. Clearly further studies are needed to clarify the discrepancies between studies and models.

In the present study, ATP12A inhibition induced a significant pH increase, at a similar level in both WT and CF airway epithelia. This points to the possible role of persistent H^+^ secretion in CF airways in ASL acidosis, in contrast to the WT cells where this secretion is counterbalanced by CFTR-dependent HCO_3_^−^ secretion, as already reported by Shah *et al*.^8^.

The fact that in mild acidic conditions in a nominally CO_2_/HCO_3_^−^ free, Hepes free-solution, ASL pH was not restored to WT values after ATP12A inhibition by ouabain demonstrates that ASL acidification in CF airways results from the coupling of persistent H^+^ secretion with a HCO_3_^−^ secretion defect. Our data show that inhibiting basolateral HCO_3_^−^ transport *via* NBC1 has a limited effect on ASL pH, suggesting that NBC1 might act indirectly as a limiting factor for HCO_3_^−^ secretion and ASL pH regulation.

We hypothesized a critical role of the CFTR-dependent HCO_3_^−^ secretion in ASL pH homoeostasis. In support, cAMP activated an electrogenic HCO_3_^−^ apical transport in the absence of Cl^−^ in WT airways, whereas this was defective in CF airways. This was indicated by IsC changes in response to cAMP in a solution depleted of Cl^−^. This confirms previous reports showing that apical HCO_3_^−^ secretion is reduced when CFTR is absent^26–28^. However, it was impossible to discern in our model any ASL pH variations in WT or F508del cells after Forskolin/IBMX treatment and CFTR inhibition in physiological conditions. The CFTR effect on pH variation was unmasked by Inh-172 CFTR-specific inhibition only in normocapnic acidosis, whereas CFTR activation by itself was not sufficient to restore pH upon mild acidosis. This suggests that ASL HCO_3_^−^ content and pH homeostasis involve transporters other than CFTR. Our results show that pendrin, an electroneutral anion exchanger, contributes to ASL pH regulation, and point to its functional defect in the context of the F508del genotype in epithelial respiratory cells^19,29–31^. Indeed, pendrin has an important role in airways for ASL hydration and has already been involved in asthma and other inflammatory airway diseases^30,32–35^.

We demonstrate for the first time pendrin dysfunction in the context of CFTR mutations and suggest a possible relevance in the field of CF physiopathology. Importantly, pendrin seems more potent than CFTR in regulating pH ASL, as its inhibitor is able to decrease pH by ~0.1 pH unit, and HCO_3_^−^ related IsC variation upon forskolin by a factor of ~2. It can be hypothesized that CFTR is important to provide the Cl^−^ ions necessary for HCO_3_^−^/Cl^−^ exchange by pendrin. Thus, lack of Cl^−^ in the context of CFTR dysfunction leads to decreased pendrin activity. Our data concur with those of Garnett *et al*., whose results suggested that pendrin regulates ASL pH by exchanging Cl^−^ with HCO_3_^−^ ^23^. Another explanation is that pendrin may also interact with CFTR with a reciprocal functional activation process, which may be defective in the context of *CFTR* mutation, similarly to what has been described for SL26A3^25^ or A9^36^. Therefore, pendrin inhibition would impact CFTR activity, including HCO_3_^−^ cAMP-dependent secretion as shown by IsC. Our results conflict with those of Haggie *et al*. who observed no significant ASL acidification in WT and F508del primary cells by pendrin activity inhibition in the absence of an inflammatory context^19,37^. This discrepancy may be related to experimental conditions and semi-quantitative pH measurements which may not have been sensitive enough to capture small variations in pH, as well as different levels of pendrin expression which is very sensitive to culture conditions and pro-inflammatory stimuli.

Taken together, our data show that abnormally low ASL pH in CF airways involves reduced HCO_3_^−^ secretion by defective CFTR and inactivated pendrin in the presence of remaining H^+^ secretion by ATP12A. Those results are however cell type-specific as recently pointed out by a recent study demonstrating that most HCO_3_^−^ secretion by Calu-3 cells was mediated by CFTR^27^.

### Defective antimicrobial capacity in CF airways is related to ASL acidification

Our study set out to investigate in the human model the pattern of initial bacterial proliferation by *S. aureus* during the first hours of infection after bacterial seeding, to gain more insight into the innate immunity of CF airways. We designed an experimental protocol to closely mimic the infection conditions in airways, including very low amounts of *S. aureus*, reflecting what naturally occurs during the initial steps of airway colonization by *S. aureus*. In these conditions, we observed a significantly increased growth of *S. aureus* in the CF epithelium. We checked that the deficient antimicrobial capacity of CF airways was exclusively related to the impaired ASL host defense as only the conditions with a very high bacteria charge detected a low level of intracellular invasion, which does not correspond to a physiological state. Most importantly, our results in the human respiratory model demonstrated that this impaired host defense is pH-dependent. Increasing CF ASL pH improved its antimicrobial capacity, and conversely, decreasing ASL pH in WT epithelium recapitulated the defective bacterial clearance of CF airways. Importantly, an ouabain-induced increase in CF ASL pH, by as little as 0.1 pH unit, was able to decrease significantly bacterial proliferation.

Moreover, inhibition of H^+^/K^+^ ATPase might be clinically relevant in situations of acidic inhalations due to gastro-esophageal reflux. Indeed we show that decreasing pH to 6.9, a value far above gastric pH (usually at 4), is associated with an enhancement of bacterial proliferation. This defect is amplified by the fact that CF cells can not compensate for mild acidic stress.This points to the potential involvement of H^+^/K^+^-ATPase in the physiopathology of initial infection, as reported by Shah *et al*.^8^.

Very importantly, we also show the potential involvement of pendrin in airway bacterial clearance, as its specific inhibition in WT airways induces a CF-like bacterial clearance defect. Pendrin seems to present a functional defect in the context of the F508del genotype. A pendrin activator that would increase HCO_3_^−^ secretion might be an interesting strategy for ASL pH regulation.

Such data also illustrate that very small variations in pH may be physiologically relevant for antimicrobial airway activity. Indeed, we observed the modulation of LL-37 and hBD1 antimicrobial activities at small pH variations, in the range of ASL pH differences observed between WT and CF epithelia. This may be explained by the modification of the electrical charge of antimicrobial peptides under small pH variations, and thus the modulation of their conformation, a key point in their antimicrobial properties^5,13,14^. The defect observed for each peptide individually may occur synergistically in ASL where peptides are expressed simultaneously.

If initiated very early, such a therapeutic strategy could therefore counteract the very first steps of the disease on pristine airways of neonates. Inhibiting *S. aureus* airway infection would then limit the pro-inflammatory cascade aggravated by infection. How ASL pH could be normalized is still unknown. As HCO_3_^−^ aerosolization effects are transient, it would be more effective to increase ASL pH sustainably. This might be achieved by various drugs that buffer mucus, even though this might modify the physiological balance of acid/base regulation of the airway epithelia. Moreover, as CF cells can not compensate for acidic challenges, control of acidic gastro-esophageal reflux is crucial. Importantly, our results suggest that pendrin activation might offer a novel strategy, by triggering ASL alkalization^38^, possibly in combination with ATP12A inhibitors^39,40^, which should be further investigated.

## Conclusion

We report evidence for defective bacterial clearance of *S. aureus* in human CF airways. Our results support that the reduced pH observed in CF ASL is central to innate immune defense. This adds a new mechanism by which impaired ASL properties in CF promote microbial infection. Our data strongly suggest that CF cells fail to properly regulate pH variations, and demonstrate a potential role for pendrin and ATP12A in pH-dependent bacterial clearance capacity. Translating this understanding into early ASL pH normalization is now an exciting challenge, with prospects for improved clinical care.

## Supplementary information

Supplemental manuscript_clean version
