# Author Correction: Airway surface liquid acidification initiates host defense abnormalities in Cystic Fibrosis

**DOI:** 10.1038/s41598-019-54253-4

**Published:** 2019-11-21

**Authors:** Juliette Simonin, Emmanuelle Bille, Gilles Crambert, Sabrina Noel, Elise Dreano, Aurélie Edwards, Aurélie Hatton, Iwona Pranke, Bérengère Villeret, Charles-Henry Cottart, Jean-Patrick Vrel, Valérie Urbach, Nesrine Baatallah, Alexandre Hinzpeter, Anita Golec, Lhousseine Touqui, Xavier Nassif, Luis J. V. Galietta, Gabrielle Planelles, Jean-Michel Sallenave, Aleksander Edelman, Isabelle Sermet-Gaudelus

**Affiliations:** 1 Inserm U1151 - CNRS UMR 8253 – Institut Necker Enfants Malades, Université Paris Sorbonne, Paris, France; 2 Centre de Recherche des Cordeliers, INSERM, , Sorbonne Université, USPC, Université Paris Descartes, Université Paris Diderot, CNRS ERL 8228, Laboratoire de Physiologie Rénale et Tubulopathies, F-75006 Paris, France; 3 Inserm, UMR1152, PR CE Université Paris Diderot, Paris, France; 4 Department of Biomedical Engineering, Boston University, Boston, United States; 5 Upres EA2511, Université Paris Descartes, Institut Pasteur, Paris, France; 6 Telethon Institute of Genetics and Medicine, Pozzuoli, Italy

Correction to: *Scientific Reports* 10.1038/s41598-019-42751-4, published online 24 April 2019

In the original version of this Article, Bérengère Villeret & Jean-Michel Sallenave were incorrectly affiliated with ‘Department of Biomedical Engineering, Boston University, Boston, United States’. The correct affiliation is listed below.

Inserm, UMR1152, PR CE Université Paris Diderot, Paris, France

In addition, Aurélie Edwards was incorrectly affiliated with ‘Inserm, UMR1152, PR CE Université Paris Diderot, Paris, France’. The correct affiliation is listed below.

Department of Biomedical Engineering, Boston University, Boston, United States

These errors have now been corrected in the HTML and PDF versions of this Article.

