# Supplementary material for: Airway surface liquid acidification initiates host defense abnormalities in Cystic Fibrosis
Source: Sci Rep. 2019 Apr 24;9:6516. doi: 10.1038/s41598-019-42751-4 (PMC6482305; doi:10.1038/s41598-019-42751-4)

**ONLINE DATA SUPPLEMENT**

# Airway surface liquid acidification initiates host defense abnormalities in Cystic Fibrosis

Juliette Simonin, Emmanuelle Bille, Gilles Crambert, Sabrina Noel, Elise Dreano, Aurélie Edwards, Aurélie Hatton, Iwona Pranke, Bérengère Villeret, Charles-Henry Cottart, Jean-Patrick Vrel, Valérie Urbach, Nesrine Baatallah, Alexandre Hinzpeter, Anita Golec, Lhousseine Touqui, Xavier Nassif, Luis J.V Galietta, Gabrielle Planelles, Jean-Michel Sallenave, Aleksander Edelman and Isabelle Sermet-Gaudelus

**Supplemental material**

**1. Human bronchial epithelial cell cultures**

Cystic fibrosis bronchial epithelial (CFBE41o-) cells, wild type (WT CFBE41o-) and F508del (F508del CFBE41o-) homozygous, were generously provided by Dr. Gruenert[1]. They were cultured in MEM/10% FBS medium (ThermoFisher Scientific, Saint Aubin, Ile-de-France France) and supplemented with 300 µg/mL hygromycin B to induce WT *vs.* F508del *CFTR* expression. Human primary bronchial epithelial (HBE) cells were obtained from lobectomies of non-CF donors and lung explants of CF patients. All the experiments were performed in accordance with the guidelines and regulations in the Declaration of Helsinki and the Huriet-Serusclat law on human research ethics.

Cells were differentiated in seeded coated Costar Transwell inserts (Sigma, Saint-Quentin-Fallavier, Auvergne-Rhône-Alpes, France) (pore size 0.4 µm, surface area 0.33 $cm^2$) at 1,000,000 cells/$cm^2$, and were then grown at an air-liquid interface (ALI) in humidified air containing 5% $CO_2$ at 37 °C, with a basal culture medium at pH 7.4 and 25 mM $HCO_3^-$ concentration, as previously described[2]. Under these experimental conditions, at 3–4 weeks post-seeding, the cell cultures formed a tight, well-differentiated polarized epithelium (transepithelial resistance > 600 $\Omega/cm^2$)[3]. Differentiation was confirmed by the presence of cilia (staining by alpha-Tubulin) and tight junctions (staining by Zona occludens-1 proteins (ZO-1). In some experiments, IL4 (10 ng/mL) was added for 2 days before experiment to over express SLC26A4.

**2. Measurement of ASL pH with a microelectrode in a controlled atmosphere**

The composition of the solutions used for experiments were followings: (i) for physiological conditions Ringer solution (equilibrated with 25 mM HCO3 /5% CO2 to obtain pH 7.4): 115 mM NaCl, 25 mM $NaHCO_3$, 2.4 mM $K_2HPO_4$, 0.4 mM $KH_2PO_4$, 1.2 mM $CaCl_2$, 1.2 mM $MgCl_2$ and 10 mM Glucose; (ii) extracellular normocapnic acidosis (equilibrated with 10 mM

HCO3 /5% CO2 to obtain pH 7.1): 130 mM NaCl, 10 mM $NaHCO_3$, 2.4 mM $K_2HPO_4$, 0.4 mM $KH_2PO_4$, 1.2 mM $CaCl_2$, 1.2 mM $MgCl_2$ and 10 mM Glucose. We checked that prolonged exposure in a 5% $CO_2$ atmosphere in this enclosure did not change the pH of the listed solutions for at least 24h.

ASL pH was measured at 37°C after incubation of the epithelium with pharmacological agonists or inhibitors of the targeted transporters. Those included: NBC-specific inhibitor S0859 (100 µM) (Adooq Bioscience, Irvine, California, United States); PKA activator forskolin (10 µM) (Sigma, Saint-Quentin-Fallavier, Auvergne-Rhône-Alpes, France) and 3-isobutyl-1-methylxanthine (IBMx) (100 µM) (Sigma, Saint-Quentin-Fallavier, Auvergne-Rhône-Alpes, France) to inhibit cAMP degradation; CFTR specific inhibitor PPQ-102 (2 µM) (generously provided by Dr. Verkman); pendrin specific inhibitor A01[4,5] (25 µM) (generously provided by Dr. Verkman); and the inhibitor ouabain (1 mM) (Sigma, Saint-Quentin-Fallavier, Auvergne-Rhône-Alpes, France).

Most of the agonists or inhibitors were applied at the basolateral side of the epithelium to avoid any potential change in ASL pH, independently of their biological effects. Only ouabain was applied apically at 1 mM to specifically target the apical ATP12A pump, as its basolateral application would inhibit the basolateral $Na^+/K^+$ ATPase.

In alternative experiments, ouabain, 1 mM, was added to the apical side of the epithelium in a nominally $CO_2/HCO_3^-$ free solution (140 mM NaCl, 2.4 mM $K_2HPO_4$, 0.6 mM $KH_2PO_4$, 1.2 mM $CaCl_2$ and 1.2 mM $MgCl_2$) while the basolateral side was bathed with a nominally $CO_2$/ $HCO_3^-$ free solution **buffered to pH 7.4** with Hepes 25 mM and containing 135 mM NaCl, 25 mM Hepes, 2.4 mM $K_2HPO_4$, 0.6 mM $KH_2PO_4$, 1.2 mM $CaCl_2$, 1.2 mM $MgCl_2$ and 10 mM Glucose.

S0859, PPQ-102 and A01 inhibitors were diluted in dimethyl sulfoxide (DMSO). DMSO induced ASL acidification which reached a decrease of 0.2 pH units after 6 hours incubation.

### 3. Quantitative RT-PCR analysis

*Acid and base transporters*

RNAs were extracted from epithelial CFBE41o- cells and bronchial primary cells cultivated on filters, with TRIzol 400 µL/filter (Invitrogen, Villebon-sur-Yvette, Ile-de-France, France) according to the manufacturer's instructions. One µg of RNAs extracted was retro-transcripted in complementary DNA (cDNA) by the RT-PCR technique (Roche Diagnostics, Meylan, Auvergne-Rhône-Alpes, France). Specific probes targeting transcripts of interest were chosen with the software Primer3Web 4.0. The cDNA was then diluted five-fold and tested by quantitative PCR with the 480 SYBR green I Master kit (Roche Diagnostics, Meylan, Auvergne-Rhône-Alpes, France) on 96-well plates. Expression for gene quantification was assessed by successive dilutions. These ranges enabled us to calculate the PCR efficiency. Normalization was based on the cyclophilin (*PPIA*) gene. Primers of the different genes are given in **Supplemental Table 1.**

*Antimicrobial peptides*

RNA was isolated from epithelial CFBE41o- cells and bronchial primary cells cultivated on filters using PureLink® RNA Mini Kit (12183018A, Ambion, Life Technologies, Saint Aubin, Ile-de-France France), following the manufacturer's instructions. Briefly, lysates were mixed with 70% ethanol and loaded onto a silica membrane column. After different washings, total RNA was eluted in DNAse-RNAse-free water and stored at −80°C until use. DNase treatment was performed prior to reverse transcription polymerase chain reaction (RT-PCR) using RNAse-free DNAse I (Roche Diagnostics, Meylan, Auvergne-Rhône-Alpes, France) at 37°C for 10 min. DNAse was then inactivated by increasing the temperature to 70°C for 10 min. cDNA was synthesized from total RNA (500 ng) using M-MLV reverse transcriptase (Promega, Madison, Wisconsin, United States) following the supplier's protocol (1 h at 37°C followed by 10 min at 70°C). Real-time quantitative PCR was performed in a total volume of

15 µL using 2x Fast SYBR® Green Master Mix (Life Technologies, Saint Aubin, Ile-de-France France), 2 µL of diluted cDNA, 2 µmol forward primer, and 2 µmol reverse primer in a 96-well plate. PCR was run with the standard program: 95°C 10 min, 40 cycles, 95°C 15s and 60°C 1 min in a 96-well plate. Results are shown as quantity of mRNA copies relative to the control condition, with *HPRT* (hypoxanthine-guanine phosphoribosyltransferase) expression used as internal control. Primers of the different genes are given in **Supplemental Table 2.**

**4. ASL antibacterial growth capacity study**

*Staphylococcus aureus* CIP 76.25 (Collection of the lnstitut Pasteur) was cultured in antibiotic-free cell culture medium. Bacterial cultures were initiated with optical density $OD_{600\ nm}$ = 0.05, and left under agitation at 37°C, 5% $CO_2$. Inoculum was prepared from bacterial culture in exponential growth phase, and the targeted inoculum concentration was obtained by appropriate dilutions of the bacterial culture. Concentration was estimated by its $OD_{600\ nm}$ and checked at the end each experiment by counting colony forming units (CFUs) after Petri dishes plating.

Epithelia were apically infected with 50 µL of different inocula. This was performed 24 hours after antibiotic removal from cell medium and 2 additional medium changes to avoid residual antibiotics. ASL was collected after cells incubation at 5% $CO_2$, 37 °C, and plated on Petri dishes. Survival bacteria were evaluated by CFUs and expressed as % from inoculum or from control condition. Presence of bacteria at the basolateral side of the infected filters was systematically checked.

To determine the effect of ASL pH conditions on bacterial growth, bacterial culture medium was set at various pH by different $HCO_3^-$ concentrations (5, 10, 25 and 40 mM $HCO_3^-$ at $pCO_2$, 5%), aiming to restore apical supernatant fluid pH in CF cultures at pH 7.45 (with the 40 mM $HCO_3^-$ concentration), and to acidify ASL pH in WT cultures at pH 6.90 (with the

5 mM $HCO_3^-$ concentration). We checked that *S. aureus* growth was not modified by different pH conditions, at 2 hours incubation starting at a 300–3,000 CFU/mL inoculum. Conversely, we checked that pH of cell culture medium was not modified by proliferation of *S. aureus* for 2 hours with an inoculum between 300 and 3,000 CFU/mL.

To evaluate inhibition of pendrin activity on bacterial growth in WT CFBE41o-, cell cultures were incubated for 6 hours at the basolateral face with 25 µM A01 *vs.* DMSO and then infected for 2 hours with an inoculum between 300 and 3,000 CFU/mL.

Adhesion and internalization capacities of *S. aureus* CIP 76.25 on the airway epithelium were evaluated by CFU counting and immunostaining after apical infection with 300–3,000 CFU/mL. For CFU counting, epithelium was infected for 2 hours, then washed with PBS to remove non-adherent extracellular bacteria. Cells were then scraped and the whole suspension was collected for CFU counting. Bacterial adhesion and internalization were evaluated overall after cell permeabilization by apical addition of sterile water. To evaluate only bacterial internalization, infected cells were incubated with 10 µg/mL membrane-impermeable lysostaphin to disrupt all extracellular bacteria just before addition of water. This enabled to differentiate between *S. aureus* internalization and adhesion.

For immunostaining experiments, infected cells were stained with 1 µM cell tracer (Life Technologies, Saint Aubin, Ile-de-France France) and fixed with 4% paraformaldehyde. *S. aureus* colonies were stained with 4 µg/mL mix of vancomycin and vancomycin- Bodipy-FL (VBFL, Life Technologies, Saint Aubin, Ile-de-France France), chosen because it specifically binds the cell wall peptidoglycan of Gram-positive bacteria and does not penetrate intact cells[6]. For internalization immunostaining experiments, cells were first incubated with 10 µg/mL lysostaphin to disrupt all extracellular bacteria. They were then stained, fixed and permeabilized with 0.1% saponin, and bacteria were stained with 4 µg/mL mix of

vancomycin and VBFL. A subset of cells treated with lysostaphin without permeabilization was labeled with mix of vancomycin and VBFL to serve as the negative control.

Filters were placed on slides for confocal microscopy reading, objective lens X60 (Leica TCS SP5 AOBS confocal microscope). Images were analyzed on 6 fields per slide with the Image J software (NIH).

Both the immunostaining and CFU counting techniques did not evidence any bacterial adhesion to the epithelium nor intracellular invasion after 2h infection with a low inoculum (300 to 3,000 CFU/mL) (3 independent experiments). Only conditions with a very high, non-physiological bacterial charge count (inoculum concentration > 10,000 CFU/mL) could detect a low level of internalization, which constituted a positive control. We never observed bacteria at the basolateral side of the filters.

## Supplementary Results

### 1 Proton flux calculations

The flux of protons was calculated in our system: surface area 0.33 $cm^2$, 50 µL of Ringer solution containing 2 acid-base buffers $HPO_4^{2-}$/ $H_2PO_4^-$ (2.4 mM, 0.4 mM) and $HCO_3^-$/$CO_2$, (25 mM/5%CO2 for pH 7.4 and 10 mM/5%$CO_2$ for pH 7.1).

The total flow of protons into the apical fluid ($F_p$, moles/s) is given by:

$$F_p = \frac{d(V[H^+])}{dt} - \frac{d(V[HCO_3^-])}{dt} - \frac{d(V[HPO_4^{2-}])}{dt} \qquad (A1)$$

V is the solution volume (50 µL), and brackets denote concentrations. The derivatives are approximated as differences over the time interval between collections.

At a given pH, $[HPO_4^{2-}]$ is determined based upon conservation of total buffer and acid-base equilibrium (pKa = 6.80):

$$pH = 6.80 + \log_{10}\left(\frac{[HPO_4^{2-}]}{[H_2PO_4^-]}\right) = 6.80 + \log_{10}\left(\frac{[HPO_4^{2-}]}{C_{PO4}^{tot} - [HPO_4^{2-}]}\right) \qquad (A2)$$

where $C_{PO4}^{tot}$ denotes the total concentration of phosphate species (2.8 mM).

Assuming that $CO_2$ and $H_2CO_3$ equilibrate very rapidly, $[HCO_3^-]$ is calculated using the Henderson-Hasselbalch equation:

$$pH = 6.09 + \log_{10}\left(\frac{[HCO_3^-]}{0.0307\, p_{CO2}}\right) \qquad (A3)$$

with $pCO_2$ maintained at 5% and in stable hygrometric concentration.

The rate of proton secretion is then divided by the area of the filter (0.33 $cm^2$).

We used this approach to calculate the proton secretion flux in the diluted ASL after application of 50 µL Ringer solution (pH = 7.4, 25 mM $HCO_3^-$, 5% $CO_2$, 37 °C) for 15-30 minutes.

Results are shown in **Supplemental Table 3**. They show that during the first 15 minutes of the experiment, a period during which transport fluxes are not yet balanced, $H^+$ fluxes are significantly increased in F508del cells, relative to WT cells. Interestingly, primary cells seem to secrete more $H^+$ than CFBE41o-. Our flux estimates are of the same order of magnitude as previously published measurements of acid-equivalent fluxes[7,8] in Calu-3 and Type II alveolar epithelial cells, namely ~ 0.1-1 µeq/hr/$cm^2$.

## REFERENCES


1. Gruenert, D. C. *et al.* Characterization of human tracheal epithelial cells transformed by an origin-defective simian virus 40. *Proc. Natl. Acad. Sci. U. S. A.* **85**, 5951–5955 (1988).
2. Pranke, I. M. *et al.* Correction of CFTR function in nasal epithelial cells from cystic fibrosis patients predicts improvement of respiratory function by CFTR modulators. *Sci. Rep.* **7**, 7375 (2017).
3. de Jong, P. M. *et al.* Ciliogenesis in human bronchial epithelial cells cultured at the air-liquid interface. *Am. J. Respir. Cell Mol. Biol.* **10**, 271–277 (1994).
4. Haggie, P. M. *et al.* Inhibitors of pendrin anion exchange identified in a small molecule screen increase airway surface liquid volume in cystic fibrosis. *FASEB J. Off. Publ. Fed. Am. Soc. Exp. Biol.* **30**, 2187–2197 (2016).
5. Schwab, A. *et al.* Functional role of Na+-HCO3- cotransport in migration of transformed renal epithelial cells. *J. Physiol.* **568**, 445–458 (2005).
6. Trouillet, S. *et al.* A novel flow cytometry-based assay for the quantification of Staphylococcus aureus adhesion to and invasion of eukaryotic cells. *J. Microbiol. Methods* **86**, 145–149 (2011).
7. Shan, J. *et al.* Bicarbonate-dependent chloride transport drives fluid secretion by the human airway epithelial cell line Calu-3. *J. Physiol.* **590**, 5273–5297 (2012).
8. DeCoursey, T. E. Hydrogen ion currents in rat alveolar epithelial cells. *Biophys. J.* **60**, 1243–1253 (1991).

## SUPPLEMENTAL TABLES

**Table 1. qRT-PCR primers for acid and base transporter quantification**

| Transporter | Oligo sequence |
|---|---|
| Pendrin | CAGGAGAGCACTGGAGGAAA |
| | CGAGGAATGTCACACAGCTG |
| ATP12A | GCACTCTCATTAACCTGCGG |
| | TTTGCTGGACTAGGATGCCA |
| AE2 | CTTCATCGCCTTCTTCCTGC |
| | AGTCACCGAGAATCCACTGG |
| NBC1 | TTCACGGAACTGGATGAGCT |
| | ACTGTGGGAGAGAAGAAGCC |

**Table 2. qRT-PCR primers for antimicrobial peptides quantification**

| Antimicrobial peptide | Oligo sequence |
|---|---|
| hBD1 | GATCATTACAATTGCGTCAGCAG |
| | CTCACTTGCAGCACTTGGCCTTC |
| hBD2 | GATGCCTCTTCCAGGTGTTTTTGG |
| | TTGTTCCAGGGAGACCACAGGTG |
| LL-37 | CGGAAATCTAAAGAGAAGATTGG |
| | TAGGGCACACACTAGGACTCTG |
| HPRT | CAGGCCAGACTTTGTTGGAT |
| | TTGCGCTCATCTTAGGCTTT |

**Table 3. Net proton flux at 15 minutes in CFBE41o- cell lines and primary cells**

| $H^+$ secretion rate (μeq/hr/$cm^2$) | WT | F508del | p |
|---|---|---|---|
| CFBE41o- | -0.65 (0.6) | 1.85 (0.36) | 0.008 |
| Primary cells | 2.4 (0.86) | 4.01 (0.29) | 0.028 |

WT: Wild type; CFBE41o- cell line. CF HBE were derived from 3 F508del homozygote donors and 1 N1303K/4005 +1G>A patient. Data are presented as mean (SEM). Comparison by nonparametric Wilcoxon test.

## SUPPLEMENTAL FIGURES

**Supplemental Figure 1. Airway surface liquid pH measurements under physiological and normocapnic acidosis conditions in WT and F508del bronchial epithelia at 15 and 30 minutes.**

Apical fluid pH was measured after apical addition of 50 µL solution for 15 and 30 minutes.

A. WT *vs.* F508del CFBE41o- cells after apical addition of 50 µL Ringer's solution (25 mM $HCO_3^-$, 5% $CO_2$). 15min: pH = 7.50 ± 0.02 *vs.* pH = 7.44 ± 0.02, $p$ = 0.008; 30min: pH = 7.49 ± 0.02 *vs.* pH = 7.38 ± 0.01, $p$ = 0.008.

B. WT *vs.* F508del CFBE41o- cells after addition of 50 µL acidic Ringer's solution (10 mM $HCO_3^-$, 5% $CO_2$). 15min: pH = 7.23 ± 0.02 *vs.* pH = 7.17 ± 0.02, $p$ = 0.01; 30min: pH = 7.18 ± 0.004 *vs.* pH = 7.11 ± 0.01, $p$ = 0.01.

For all conditions, n=3 in triplicate. Data are presented as mean ± SEM. Statistical significance from unpaired nonparametric Wilcoxon test. *: $p<0.05$; **: $p<0.01$.

**Supplemental Figure 2. RTqPCR quantification of main acids and bases transporters in WT *vs.* F508del CFBE41o- and human bronchial epithelial primary cells.**

mRNA expressed in ratio from the WT condition, of ATP12A, AE2, SLC26A4 and NBC1 in WT (grey box) and F508del (black box) cells. Upper panel: CFBE41o$^-$; n=3, 6 replicates per experiments. Lower panel: human bronchial epithelial (HBE) primary cells, n=1, 4 replicates.

Data are presented as mean ± SEM. Comparison with the t-test. *: p<0.05; **: p<0.01; NS: non significant.

**Supplemental Figure 3. Airway surface liquid pH measurements upon ATP12A inhibition in acidic conditions in WT and F508del CFBE41o- cells.**

CFBE41o- were incubated for 2 hours in a nominally $CO_2/HCO_3^-$ free solution which was buffered with Hepes at the basal side (pH=7.4, 0 mM $HCO_3^-$, 25 mM Hepes, 0% $CO_2$) containing Forskolin/IBMX 10 µM/100 µM and a Hepes free-solution at the apical side (pH=7.25**,** 0 mM $HCO_3^-$, 0 mM Hepes, 0% $CO_2$). Ouabain 1mM (or Ringer as vehicle) was added at the apical side for 2 hours, as ATP12A inhibitor.

Apical fluid pH was measured after 2 hours incubation with 1 mM ouabain (hatched bars) or not (empty bars).

A. WT CFBE41o- cells. pH = 6.95 ± 0.02 (Ringer) *vs.* pH = 7.10 ± 0.04 (Ouabain), $p$ = 0.01.

B. F508del CFBE41o- cells. pH = 6.74 ± 0.05 (Ringer) *vs.* pH = 6.86 ± 0.01 (Ouabain), $p$ = 0.01.

For all conditions, n=3 evaluated in triplicate. Data are presented as mean ± SEM. Statistical significance from unpaired nonparametric Wilcoxon test. *: $p$<0.05.

**Supplemental Figure 4. Airway surface liquid pH measurements upon NBC1 inhibition in WT and F508del CFBE41o- cells.**

Apical fluid pH was measured after 2 hours incubation of 50 µL Ringer's solution (25 mM $HCO_3^-$, 5% $CO_2$) and addition of DMSO (empty bars) or 100 µM S0859 NBC inhibitor (hatched bars) for 10 minutes.

A. WT CFBE41o- cells. pH = 7.39 ± 0.01 (DMSO) *vs.* pH = 7.36 ± 0.01(S0859), $p$ = 0.008.

B. F508del CFBE41o- cells. pH = 7.24 ± 0.01 (DMSO) *vs.* pH = 7.25 ± 0.01 (S0859), NS.

For all conditions, n=3 in triplicate. Data are presented as mean ± SEM. Statistical significance from unpaired nonparametric Wilcoxon test. *: $p$<0.01; NS: non significant.

**Supplemental Figure 5. Airway surface liquid pH measurements time course under physiological and normocapnic acidosis conditions upon cAMP-dependent activation in WT and F508del CFBE41o- cells.**

WT CFBE41o- cells were incubated at the basal side with 10 µM Forskolin + 100 µM IBMX (hatched bars) *vs*. solvent (DMSO + ethanol) (empty bars). ASL pH was measured after apical addition of 50 µL physiological Ringer's solution (25 mM $HCO_3^-$, 5% $CO_2$) (*i.e.*, physiological conditions) or acidic Ringer's solution (10 mM $HCO_3^-$, 5% $CO_2$) (*i.e.*, acidic conditions). For all measurements, co-incubation of Ringer's and Forskolin+IBMX *vs.* solvent.

A. Physiological conditions (Control *vs.*, Forskolin/IBMX). 15min: pH = 7.50 ± 0.02 *vs.* pH = 7.46 ± 0.01, NS; 30min: pH = 7.49 ± 0.02 *vs.* pH = 7.49 ± 0.01, NS; 1h: pH = 7.47 ± 0.02 *vs.* pH = 7.49 ± 0.01, NS; 2h: pH = 7.46 ± 0.01 *vs.* pH = 7.49 ± 0.01, NS; 6h: pH = 7.42 ± 0.02 *vs.* pH = 7.43 ± 0.01, NS. For all conditions, n=2 in triplicate.

B. Acidic conditions (Control *vs.*, Forskolin/IBMX).. 15min: pH = 7.09 ± 0.03 *vs.* pH = 7.08 ± 0.02, NS; 30min: pH = 7.13 ± 0.03 *vs.* pH = 7.10 ± 0.03, NS; 1h: pH = 7.10 ± 0.04 *vs.* pH = 7.07 ± 0.03, NS; 2h: pH = 7.10 ± 0.02 *vs.* pH = 7.11 ± 0.05, NS; 6h: pH = 7.15 ± 0.08 *vs.* pH = 7.05 ± 0.02, NS. For all conditions, n=1 in triplicate.

Data are presented as mean ± SEM. Statistical significance from unpaired nonparametric Wilcoxon test. NS for all the comparisons.

**Supplemental Figure 6. Airway surface liquid pH measurements upon CFTR inhibition with PPQ-102 in WT and F508del CFBE41o- cells.**

WT and F508del CFBE41o- pH was measured after apical addition of 50 µL Ringer (25 mM $HCO_3^-$, 5%$CO_2$) and DMSO (empty bars) *vs.* 2 µM PPQ-102 (hatched bars) .

A. WT CFBE41o-. pH = 7.40 ± 0.02 *vs.* pH = 7.39 ± 0.02, NS.

B. F508del CFBE41o-. pH = 7.21 ± 0.01 *vs.* pH = 7.20 ± 0.01, NS.

Data are presented as mean ± SEM. n = 2 in triplicate. Statistical significance from unpaired nonparametric Wilcoxon test. NS for all the comparisons.

**Supplemental Figure 7. Airway surface liquid pH measurements under physiological and normocapnic acidosis conditions upon CFTR inhibition in WT CFBE41o- cells at 30 minutes and 2 hours incubation.**

pH was measured after addition at the apical side of 50 µL physiological Ringer's solution (25 mM $HCO_3^-$, 5% $CO_2$,) (upper panel) or 50 µL acidic Ringer's solution (10 mM $HCO_3^-$, 5% $CO_2$) (lower panel) for 30 minutes or 2 hours. WT CFBE41o- cells were incubated at the basal side with 10 µM Forskolin + 100 µM IBMX + 5 µM Inh-172 (hatched bars) *vs.* solvent (DMSO + ethanol) (empty bars).

A. Physiological conditions At 30min: pH = 7.40 ± 0.01 (control) *vs.* pH = 7.39 ± 0.003 (Forskoline), NS.

B. Physiological conditions.. At 2h: pH = 7.38 ± 0.00 (control) *vs.* pH = 7.38 ± 0.003 (Forskoline), NS.

C. Acidic conditions. At 30min: pH = 7.08 ± 0.02 (control) *vs.* pH = 7.03 ± 0.01 (Forskoline), *p* = 0.008.

D. Acidic conditions. At 2h: pH = 7.08 ± 0.02 (control) *vs.* pH = 7.02 ± 0.02 (Forskoline), *p* = 0.008.

For A and B, n= 1 in triplicate. For C and D, n=3 in triplicate. Data are presented as mean ± SEM. Statistical significance from unpaired nonparametric Wilcoxon test. *: $p<0.05$; NS: non significant.

**Supplemental Figure 8. Summary of short-circuit current measurements under 0 Chloride conditions and SLC26A4 inhibition in human bronchial epithelial primary cells.**

Experiments were done in WT human bronchial epithelial (HBE) primary cells bathed with a solution with 0 $Cl^-$ and 25 mM $HCO_3^-$, bubbled with 95% $O_2$ and 5% $CO_2$ at the apical and basal faces. Short-circuit current variation are induced by successive addition of amiloride (100 µM), Forskolin/IBMx (10 µM/100 µM), VX770 (10 µM), Inh-172 (5 µM). Cells were incubated with IL4 (10 ng/mL) for 2 days before experiment to increase SLC26A4 expression.

A. Representative tracings in control condition.

B. Representative tracings after A01 (25 µM) incubation for 6 hours before experiments.

C. Summary of results obtained from 3 WT subjects (4 independent experiments, 7 filters) in control conditions (empty bars) and after A01 incubation (hatched bars). ΔForskolin/IBMX: 5.60 ± 0.82 µA/$cm^2$ *vs.* 2.83 ± 0.55 µA/$cm^2$ , $p$=0.02; ΔInh-172: -1.90 ± 0.30 µA/$cm^2$ *vs.* -1.14 ± 0.13 µA/$cm^2$, $p$=0.03.

Data are presented as mean ± SEM. Statistical significance from unpaired nonparametric Wilcoxon test. *: $p<0.05$.

**Supplemental Figure 9. Airway Surface Liquid bacterial killing capacity in WT CFBE41o- and Human Bronchial Epithelial primary cells after *Staphylococcus aureus* CIP 76.25 apical infection according to inoculum and infection time point.**

Epithelia were infected with 50 µL inoculum of *S. aureus* CIP 76.25. ASL was collected at the end of the experiments and plated on Petri dishes to count the survival bacteria, expressed in % from inoculum under different conditions.

A. WT CFBE41o- cells infected with different inoculum concentration for 2h: 300 CFU/mL (n=4 in triplicate); 3,000 CFU/mL (n=4 in triplicate), NS.

B. WT human bronchial epithelial (HBE) primary cells infected with different inoculum concentration for 2h: 300 (n=2 in triplicate); 3,000 CFU/mL (n=9 in triplicate), $p = 0.04$.

C. WT CFBE41o- cells infected with 300 CFU/mL. ASL collection at different time points: 2h (n=4 in triplicate); 4h (n=4 in triplicate); 6h (n=2 in triplicate), $p = 0.003$.

D. WT HBE primary cells infected with 3,000 CFU/mL. ASL collection at different time points: 2h (n=9 in triplicate); 4h (n=2 in triplicate); 6h (n=8 in triplicate), $p = 0.0007$.

Data are presented as mean ± SEM. Statistical significance from unpaired nonparametric Wilcoxon test (A and B) and from unpaired nonparametric Kruskal-Wallis test (C and D). *: $p<0.05$; **: $p<0.01$; ***: $p<0.001$; NS: non significant.

**Supplemental Figure 10. Comparison of Airway Surface Liquid bacterial killing capacity in WT and F508del CFBE41o- and Human Bronchial Epithelial primary cells after *Staphylococcus aureus* CIP 76.25 apical infection.**

Epithelia were apically infected with *S. aureus* CIP 76.25. ASL was collected at the end of the experiments and plated on Petri dishes to count the survival bacteria expressed in % from the inoculum.

A. CFBE41o- cells. 300 CFU/mL inoculum. % of survival bacteria at 2h: 70 ± 12% in WT *vs.* 112 ± 15% in F508del, n=5 in triplicate, $p = 0.01$.

B. Human Bronchial Epithelial primary cells. 3,000 CFU/mL inoculum. % of survival bacteria at 6h: 3764 ± 1471% in WT (n=8 in triplicate) *vs.* 78861 ± 25170% in F508del, (n=2 in triplicate), $p = 0.04$.

Data are presented as mean ± SEM. Statistical significance from unpaired nonparametric Wilcoxon test. *: $p<0.05$; **: $p=0.01$.

**Supplemental Figure 11. Adhesion evaluation after apical infection with *S. aureus* CIP 76.25 in airway surface liquid of WT and F508del CFBE41o- cells.**

Epithelia were apically infected with 3,000 CFU/mL of *S. aureus* CIP 76.25 for 2h. CFBE41o- cells were labelled with Red cell tracker (red) and *S. aureus* was labelled with fluorescent vancomycin (green). Field surface: 0.02 $cm^2$.

A. WT CFBE41o- cells.

B. F508del CFBE41o- cells.

**Supplemental Figure 12. Effect of SLC26A4 inhibition on clearance of *S. aureus* CIP 76.25 in airway surface liquid of WT CFBE41o$^-$.**

WT CFBE41o- were incubated for 6 hours with 25 µM of the SLC26A4 inhibitor A01 (hatched bars) *vs.* DMSO vehicle (empty bars) at the basal face and apically infected for 2 hours. ASL was collected at the end of the experiments and plated on Petri dishes to count survival bacteria, expressed as ratio to the DMSO condition.

WT CFBE41o-, % of survival bacteria from DMSO: 176 ± 16%, n=2 in triplicate, $p = 0.02$.

Data are presented as mean ± SEM. Statistical significance from unpaired nonparametric Wilcoxon test. *: $p<0.01$.

**Supplemental Figure 13. qRT-PCR quantification of antimicrobial peptides in WT *vs.* F508del CFBE41o- cells and bronchial epithelial primary cells at basal condition and after *S. aureus* infection.**

mRNA levels of hBD1, hBD2 and LL-37 in WT and F508del bronchial primary cells and CFBE41o- cells under basal condition and after 3,000 CFU/mL 2h infection with *S. aureus* CIP 76.25.

For all conditions: n=3 in triplicate. Data are presented as mean ± SEM. Statistical significance from unpaired nonparametric Wilcoxon test. NS: non significant.

**Supplemental Figure 14. qRT-PCR quantification of antimicrobial peptides in WT CFBE41o- cells after incubation at different luminal pH values.**

mRNA levels of hBD1, hBD2 and LL-37 in ASL collected from WT and F508del CFBE41o- cells incubated for 24h at pH 6.9 (5 mM $HCO_3^-$), 7.4 (25 mM $HCO_3^-$) and 7.6 (40 mM $HCO_3$).

For all conditions: n=2, 4 filters per experiment. Data are presented as mean ± SEM. Statistical significance from unpaired nonparametric Wilcoxon test. NS: non significant.

Supplemental Figure 1

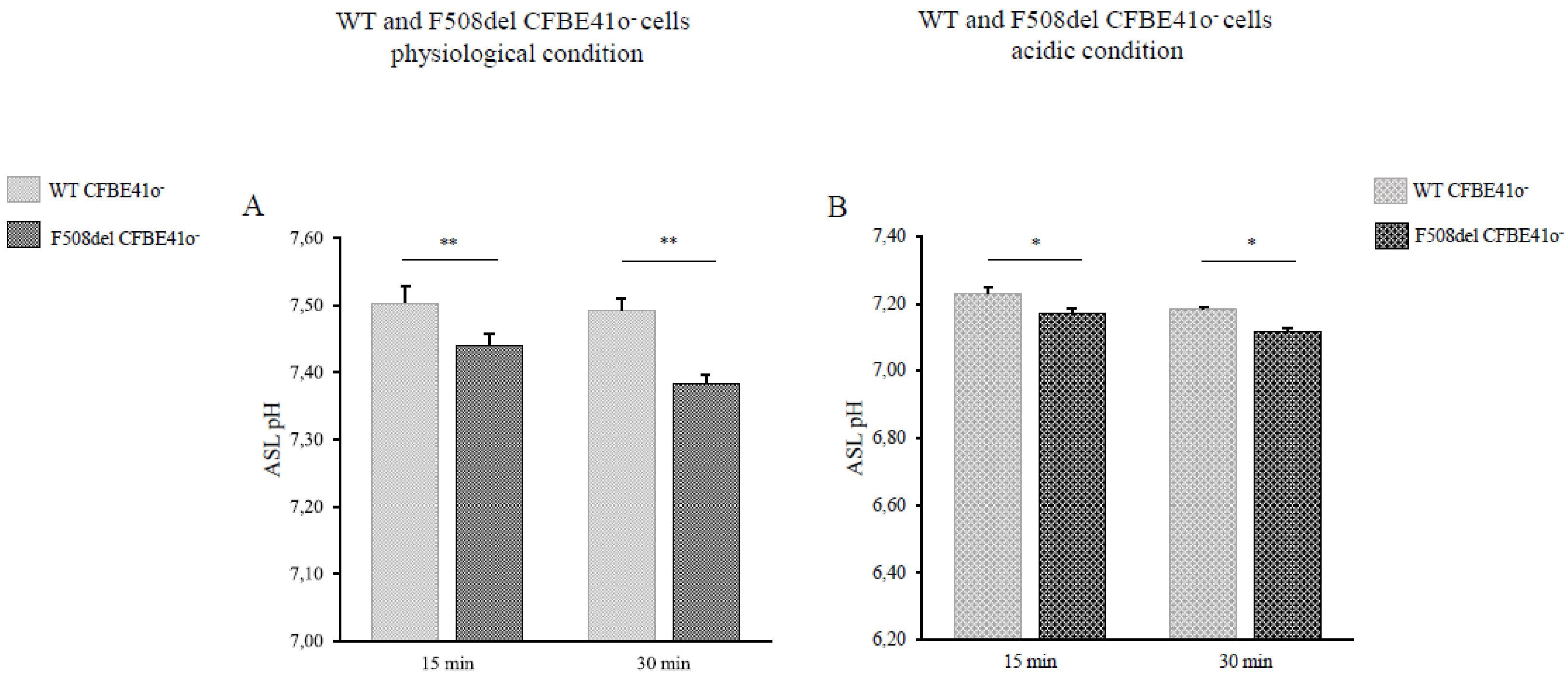

Supplemental Figure 2

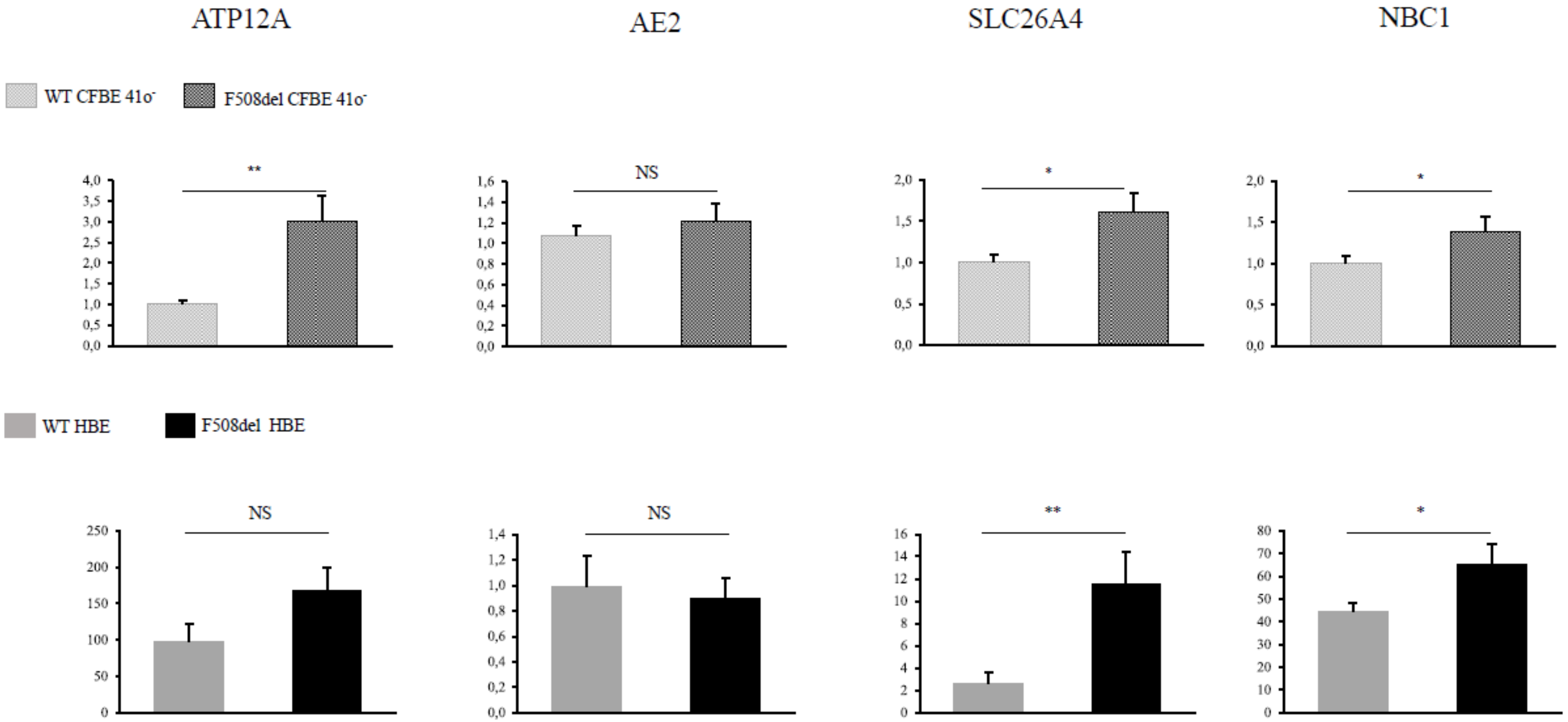

Supplemental Figure 3

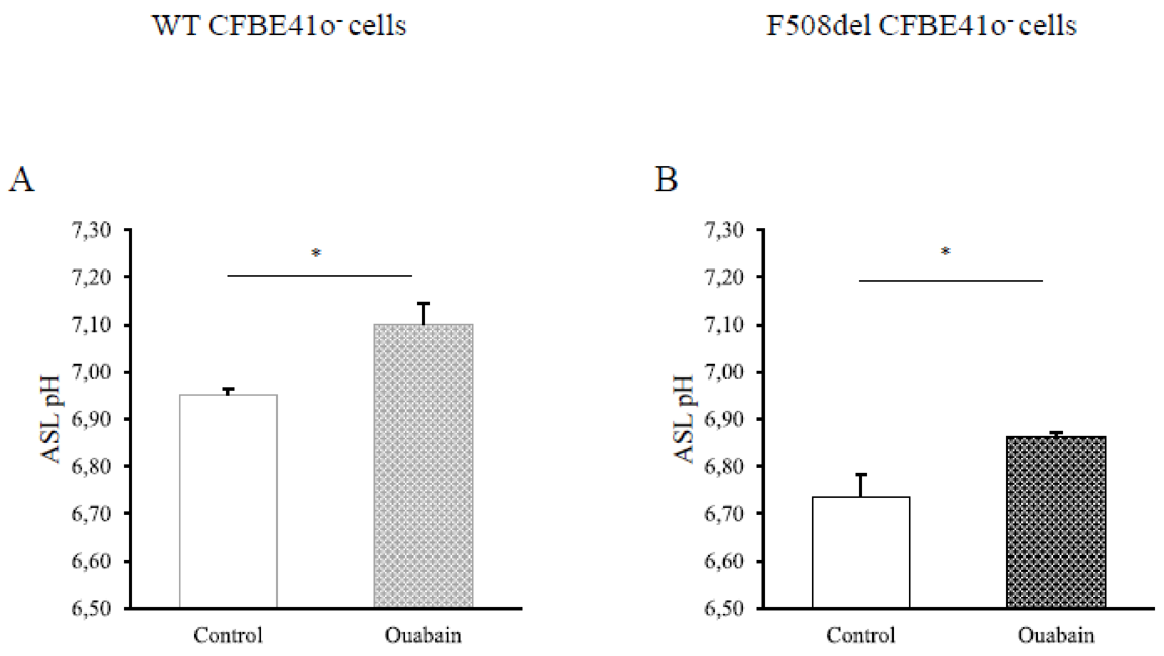

Supplemental Figure 4

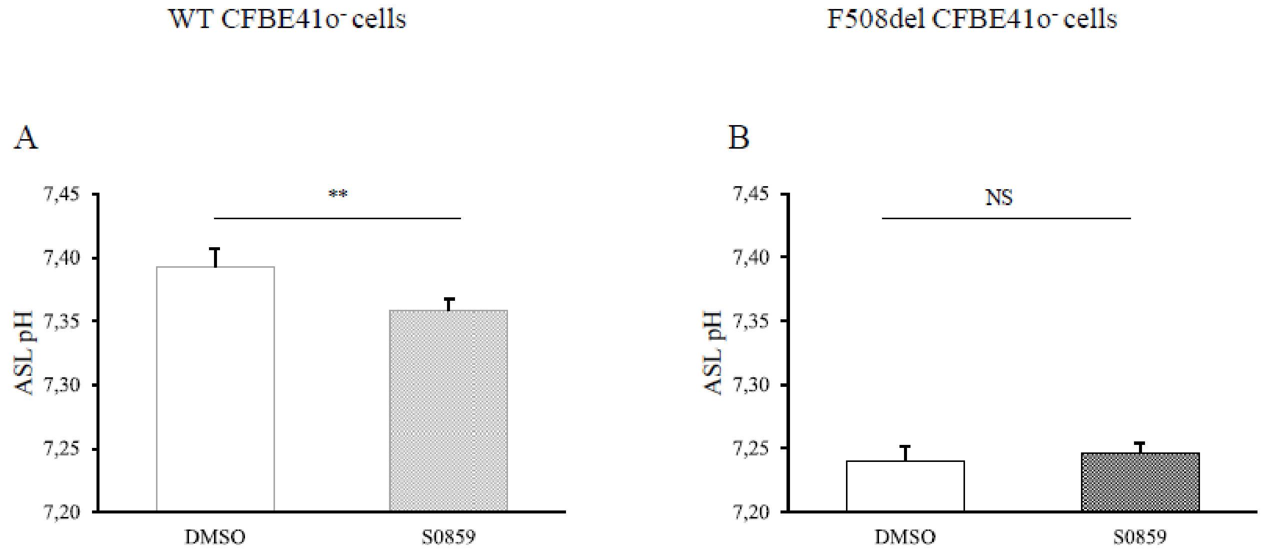

Supplemental Figure 5

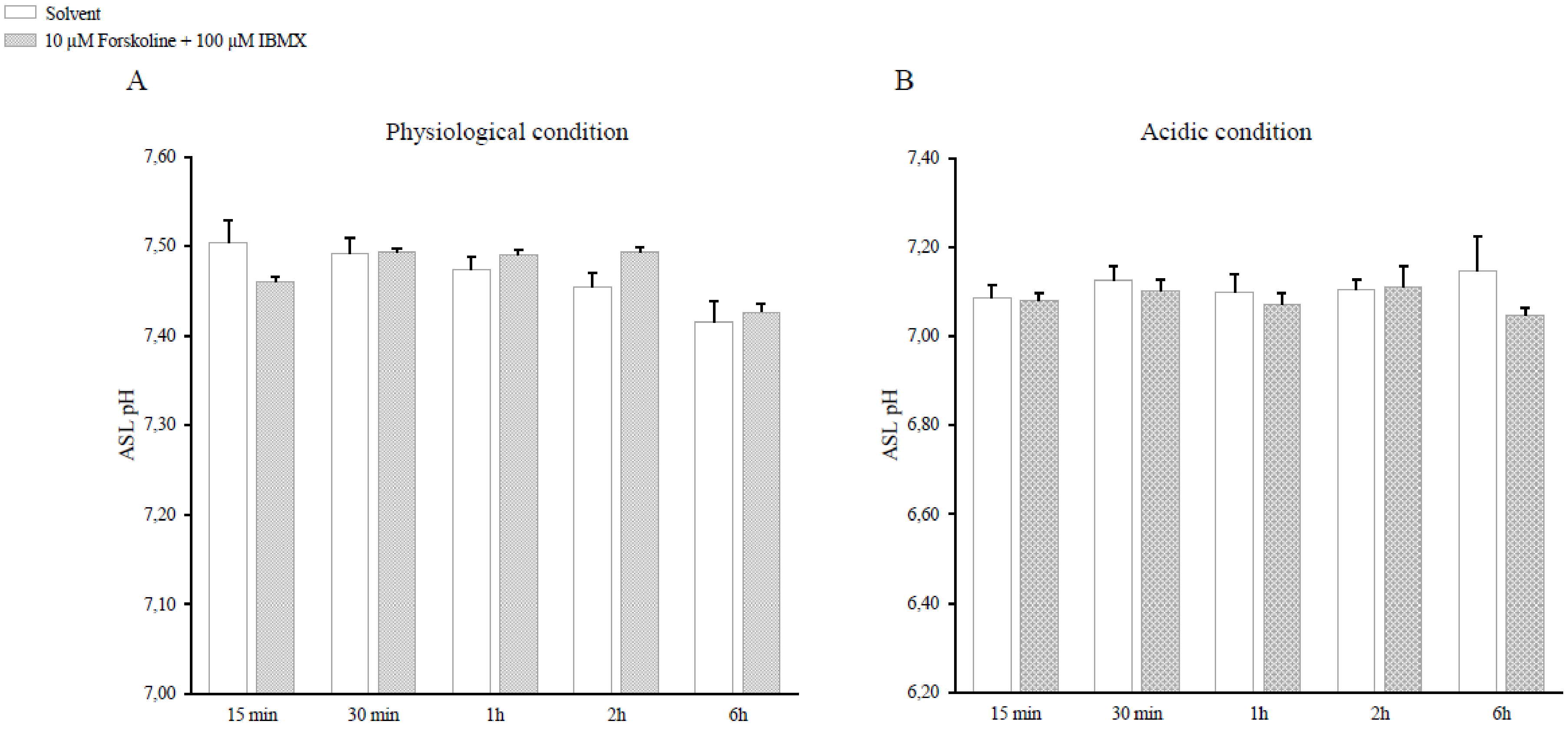

Supplemental Figure 6

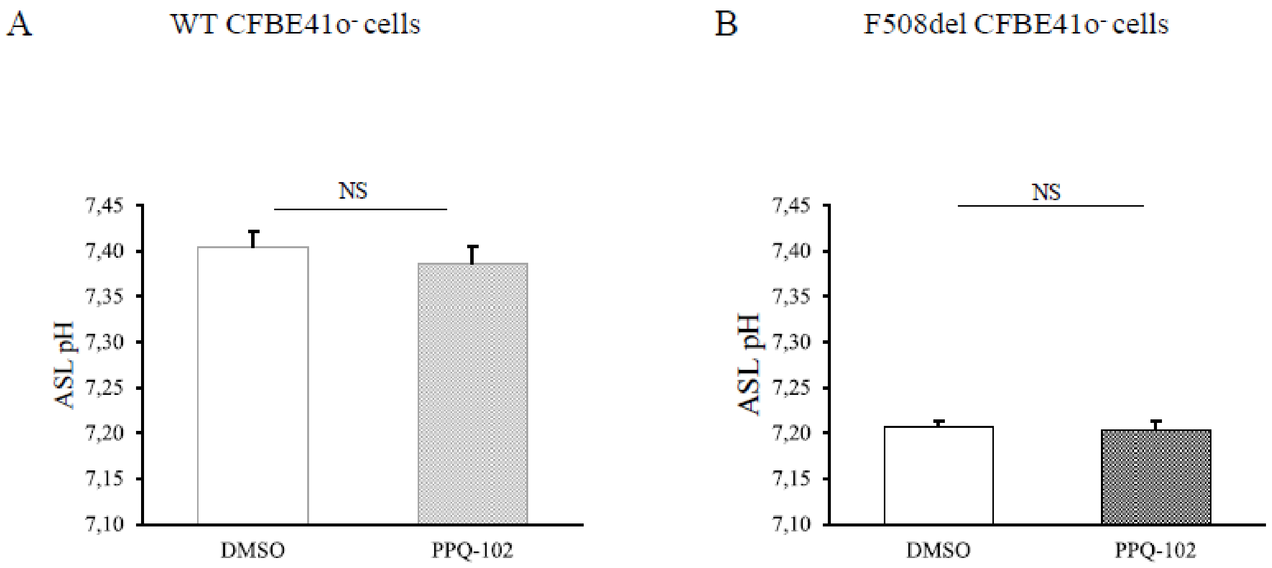

Supplemental Figure 7

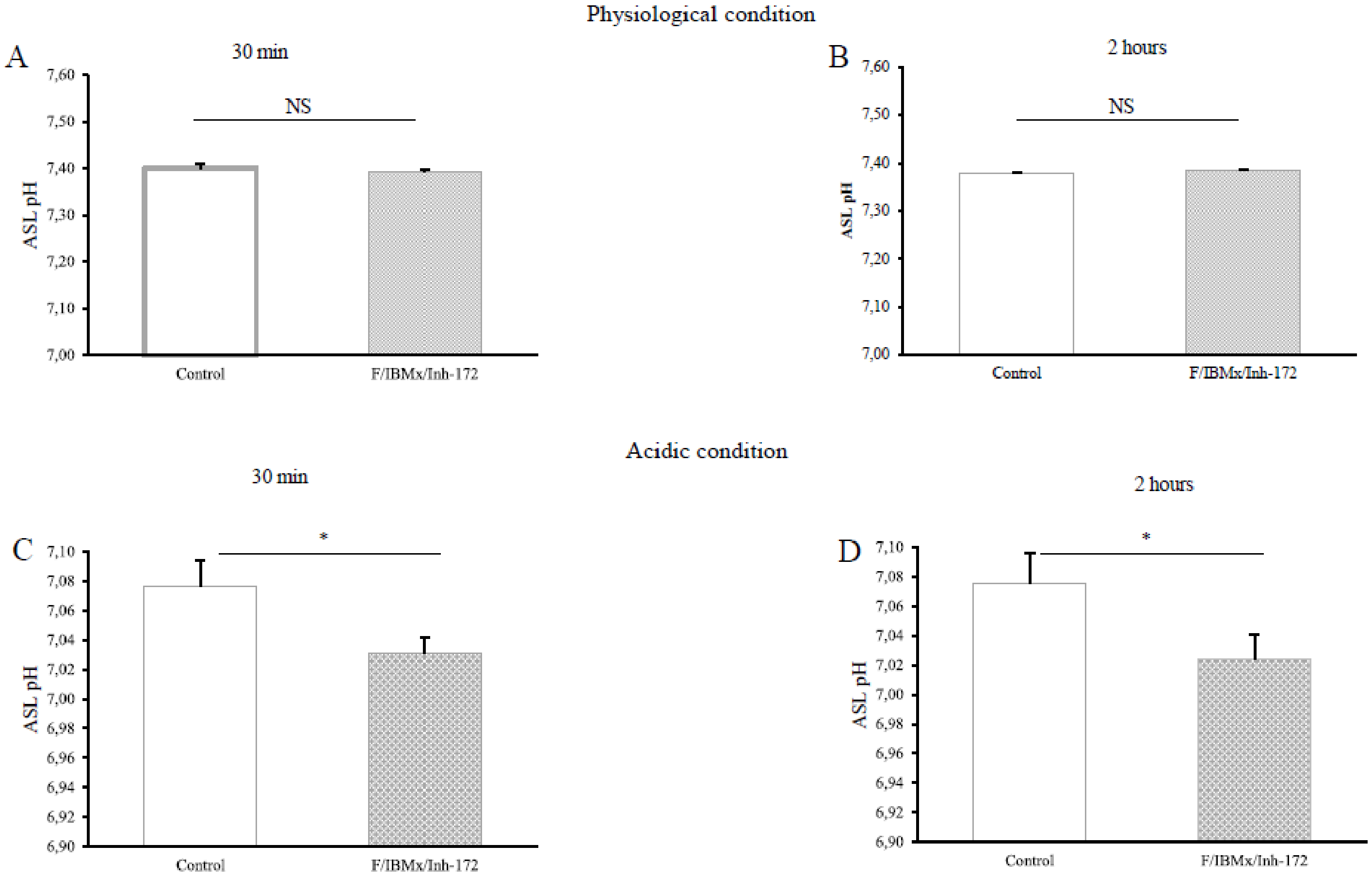

Supplemental Figure 8

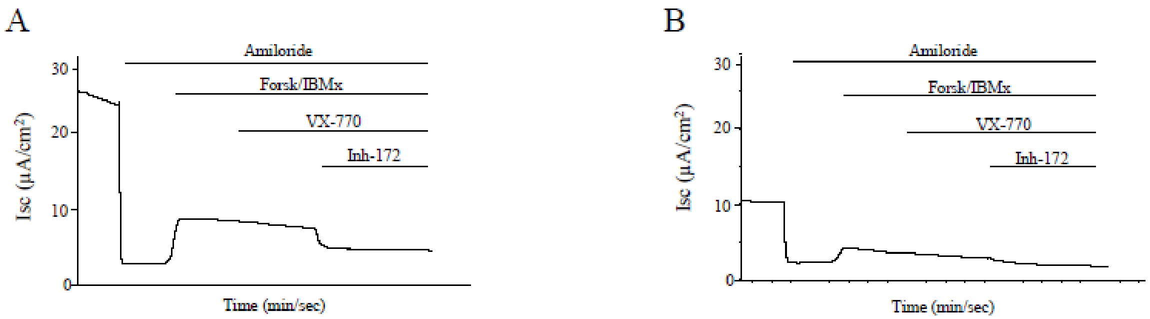


Summary of Short-circuit Current experiments in WT/CF HBE cells (0 Chloride conditions)

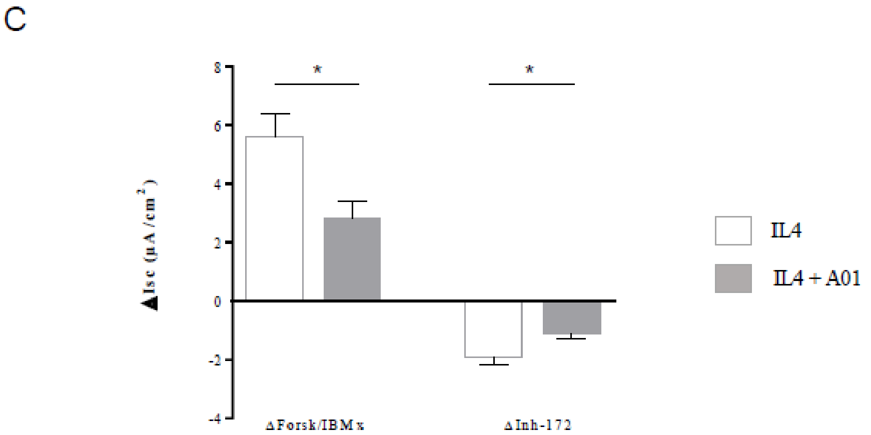

Supplemental Figure 9

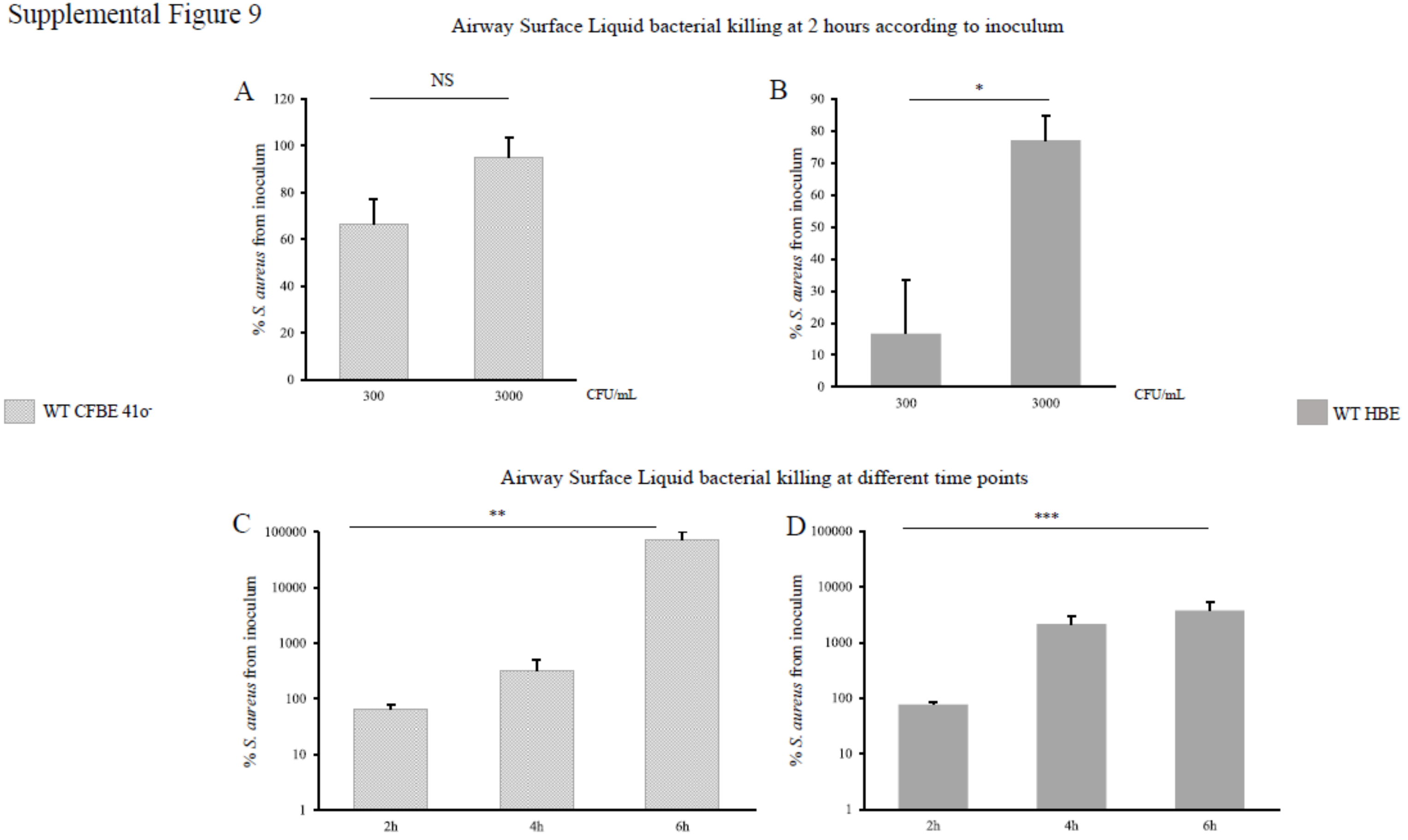

Supplemental Figure 10

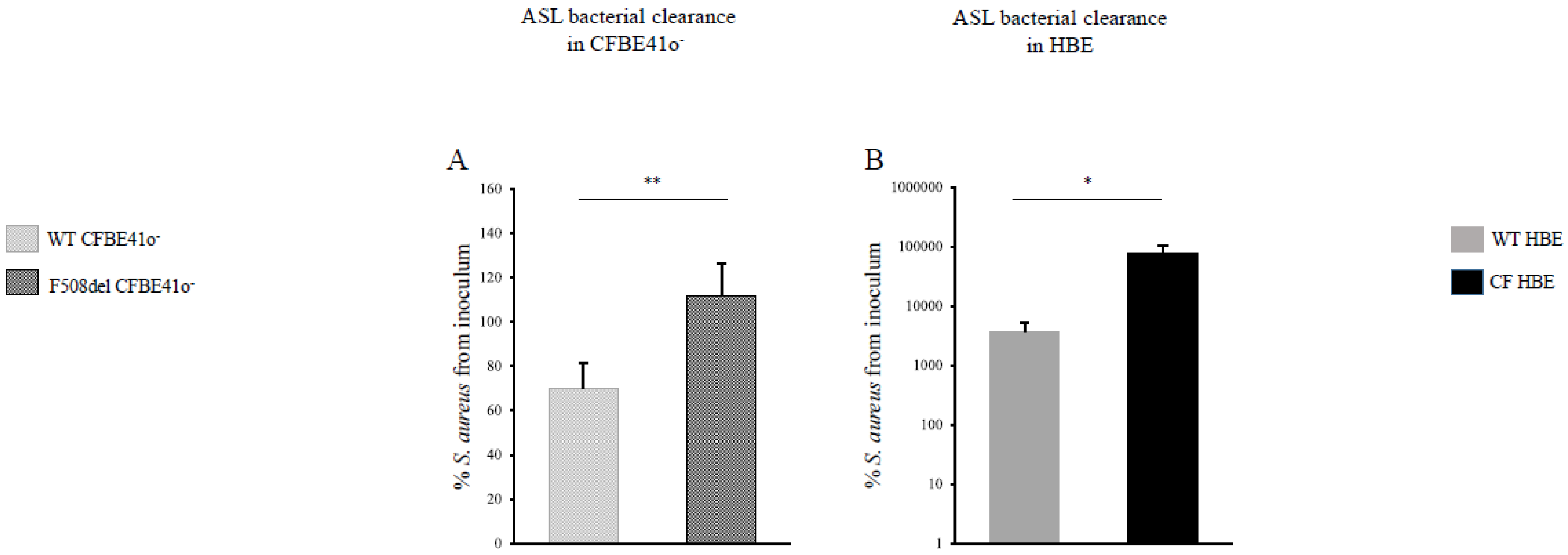

Supplemental Figure 11

A B

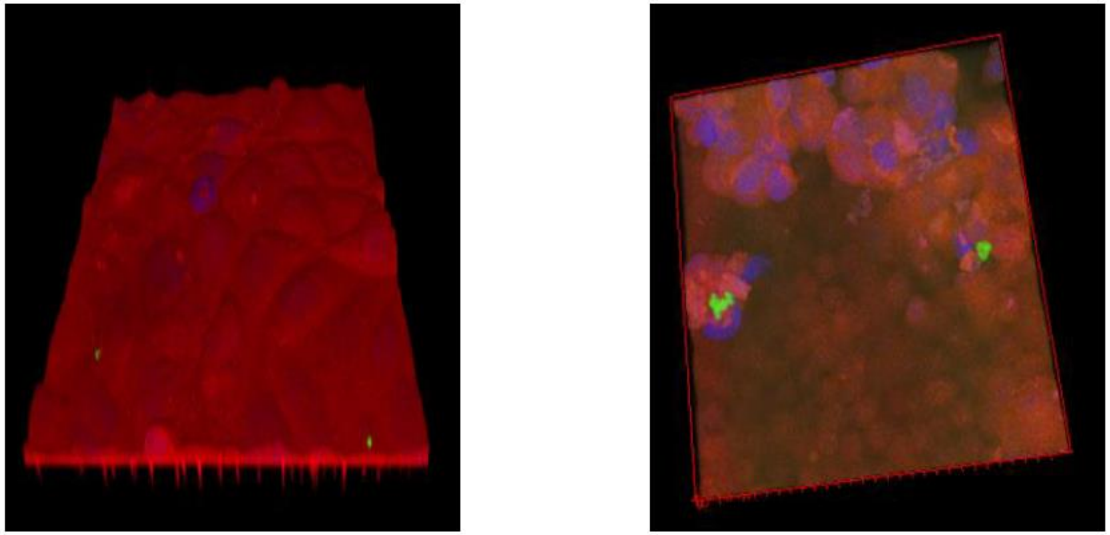

Supplemental Figure 12

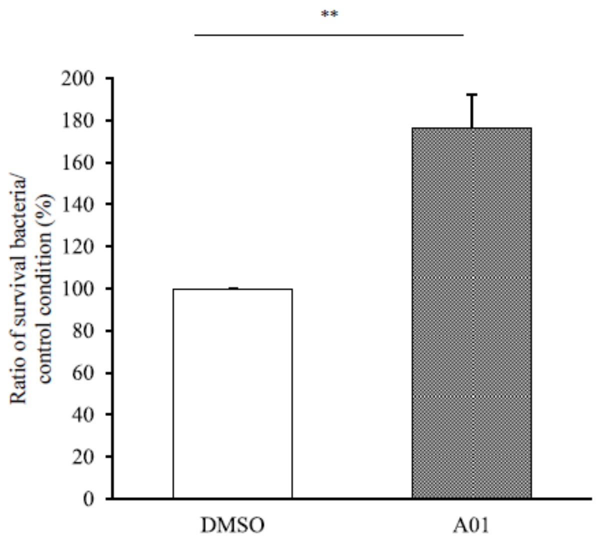

Supplemental Figure 13

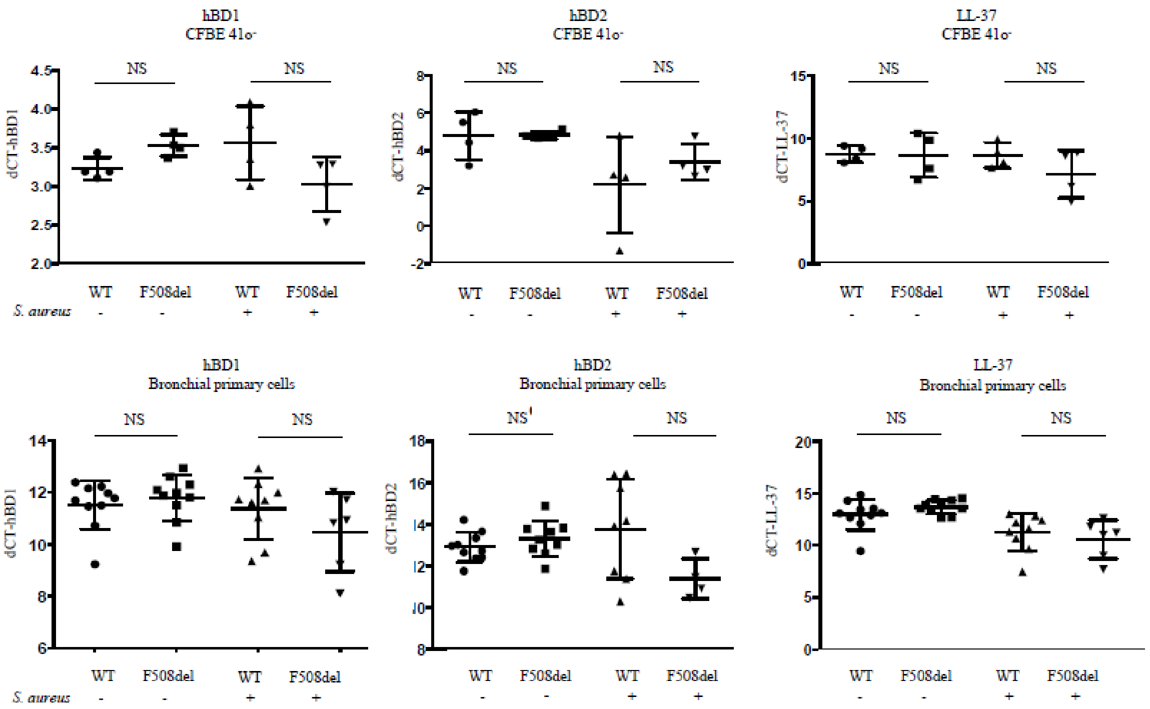

Supplemental Figure 14

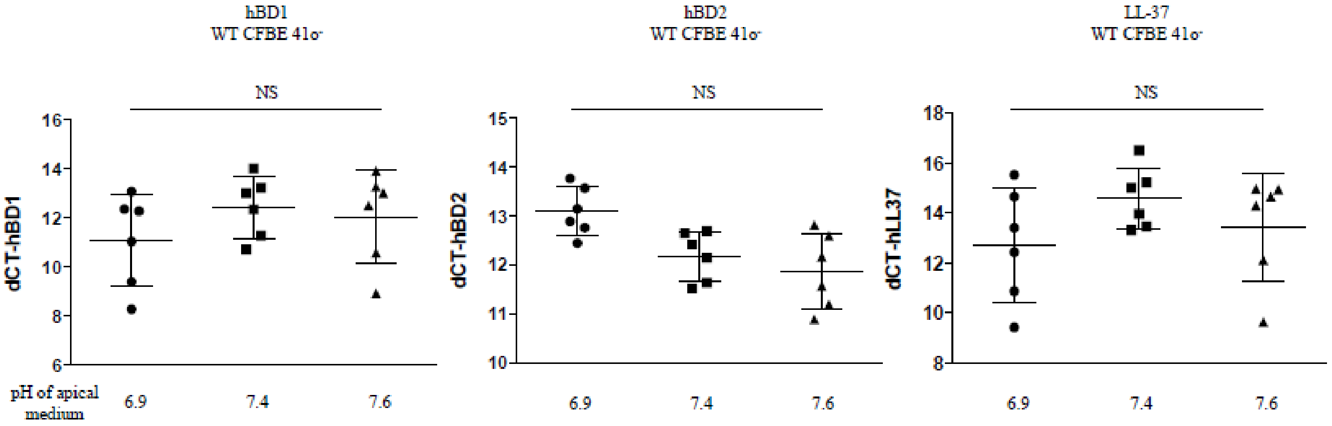

Supplement: Supplementary file 1 — Supplemental manuscript_clean version [file 41598_2019_42751_MOESM1_ESM.pdf]
